# Runtime verification of embedded real-time systems

**DOI:** 10.1007/s10703-013-0199-z

**Published:** 2013-11-07

**Authors:** Thomas Reinbacher, Matthias Függer, Jörg Brauer

**Affiliations:** 1 Embedded Computing Systems Group, Vienna University of Technology, Treitlstrasse 3, 1040 Vienna, Austria; 2 Embedded Software Laboratory, RWTH Aachen University and Verified Systems International GmbH, Am Fallturm 1, 28359 Bremen, Germany

**Keywords:** Runtime verification, Embedded real-time systems, Past-time logics, Online monitoring

## Abstract

We present a runtime verification framework that allows on-line monitoring of past-time Metric Temporal Logic (ptMTL) specifications in a discrete time setting. We design observer algorithms for the time-bounded modalities of ptMTL, which take advantage of the highly parallel nature of hardware designs. The algorithms can be translated into efficient hardware blocks, which are designed for reconfigurability, thus, facilitate applications of the framework in both a prototyping and a post-deployment phase of embedded real-time systems. We provide formal correctness proofs for all presented observer algorithms and analyze their time and space complexity. For example, for the most general operator considered, the time-bounded Since operator, we obtain a time complexity that is doubly logarithmic both in the point in time the operator is executed and the operator’s time bounds. This result is promising with respect to a self-contained, non-interfering monitoring approach that evaluates real-time specifications in parallel to the system-under-test. We implement our framework on a Field Programmable Gate Array platform and use extensive simulation and logic synthesis runs to assess the benefits of the approach in terms of resource usage and operating frequency.

## Introduction

Rigorous verification strategies are especially vital for the domain of safety-critical embedded real-time systems [48] where systems often do not only need to comply with a set of functional requirements but also—equally important—with tight timing constraints. Correct behavior of these systems is defined by the sequence of data they produce—either internally or at their physical outputs—complemented with their temporal behavior. The key idea behind formal verification techniques such as model checking [6, 22] is to exhaustively *check all executions* of a structure that is related to an implementation and its environment against given requirements, the latter of which are often formalized in terms of a temporal logic. Exhaustive analysis of programs, however, often suffers from practical infeasibility (due to state space explosion [21]) and/or theoretical impossibility (due to undecidability results).

In runtime verification [9], observers are synthesized to automatically evaluate the *current execution* of a system-under-test (SUT), typically from a formal specification in a logic that is suitable to cover certain forms of real-world specifications. The on-the-fly nature of runtime verification can be coupled with costly overhead [10, 56, 71]. Some mitigated overhead by reducing instrumentation points [34]; others ported the system and/or the observers to a more powerful architecture, such as database systems [8]. These artifacts of runtime verification are not compatible with embedded real-time systems running on ultra-portable hardware with power and performance limitations [65].

To evaluate specifications, runtime verification depends on observations of the state of the SUT. These observations are referred to as events and are input to the observer. However, the SUT’s state typically is not directly observable.

An approach classically taken in runtime verification to obtain observations is to instrument the code base, a technique that has proven feasible for a number of high-level implementation languages such as C, C++, and Java [9, 39, 40, 64] as well as for hardware description languages such as VHDL and Verilog [4, 77]. Instrumentation can be done manually, or automatically by scanning programs for assignments and function calls at the level of the implementation language and then inserting hook-up functions that emit relevant events to an observer. However, for the domain of (safety-critical) embedded real-time systems, existing approaches, despite the considerable progress in the past, are not directly applicable; mainly due to the following limitations:

- Source code instrumentation of high-level languages can only capture events that are accessible from within the instrumented software system. Embedded systems [59] often include both hardware and mechanical parts; events from those might go unnoticed for an instrumenting runtime verification approach.
- The timing behavior of the SUT is altered by instrumentation [23, 34]. The additional runtime overhead may drastically impact the correctness of a heavy-loaded real-time application with tight deadlines. The same applies to memory consumption of resource constrained systems. The relevance of this argument is supported by the fact that restricted architectures are often used in critical environments[12, 33, 66], such as in nuclear power plants [28] and spacecrafts [30, Chap. 3].
- Instrumentation may make re-certification of the system onerous (e.g., systems certified for civil aviation after DO-178B [73]).
- In its present shape, runtime verification often analyzes the correctness of high-level code. However, to show that a high-level specification is correctly reproduced by the target system, it is further necessary to show the correctness of the translation of the high-level code into executable code, i.e., the compiler. Despite recent breakthroughs [52, 53], only few verified compilers are used in practice and flaws introduced by compilers [31, 55, 81] may remain undetected by existing approaches.
- Instrumentation at binary code level may circumvent the process of establishing correctness of the compiler. However, binary instrumentation is incomplete as long as a sound reconstruction of the control flow graph is not obtained from the binary. Despite being an active area of research [7, 35, 46, 67], generating sound yet precise results remains a challenge.

There exist, however, systems and applications [80], where the relevant events can be observed without the need to infuse additional functions into the high-level code. Consider, for example, an implementation of a network protocol, where the task is to check the correctness of data flow between two network nodes. It appears natural to place an additional (passive) node in the network that collects events sent over the network, rather than instrumenting the high-level code of the network nodes. The strength of an approach like this is that collecting of events is non-intrusive, at least, as long as the additional node is passive and does not actively participate in the communication. It is important to observe that information exchange among systems is often performed by standardized interfaces. This is especially the case for embedded real-time systems, at various levels of detail [59, Chap. 3]. For certain systems, wiretapping is the only option left to gain information of the state of the system, for example, if the design includes proprietary hardware or software components.

**Fig. 1:**
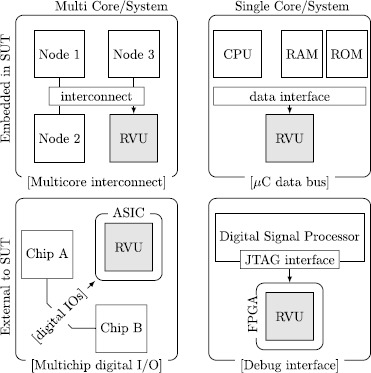
Sample applications of an instance of the proposed framework, i.e., the Runtime Verification Unit (RVU). *Top left*: RVU embedded into a network-on-chip, monitoring data exchanged among network nodes; *Top right*: RVU connected to the data interface of a microcontroller IP-core, monitoring microcontroller behavior (software); *Bottom left*: RVU connected to digital interconnects among chips on a printed circuit board (PCB), monitoring data exchanged through digital I/Os; *Bottom right*: RVU running on an FPGA attached to a debug interface of a digital signal processor, monitoring changes of accessible registers and diagnosis indicators

In the light of the discussion above, we proceed by defining requirements of a runtime verification framework targeting embedded real-time systems. We aim at a framework that is transparent to a hardware implementation, so as to be attached to or embedded into various SUTs. Examples of applications are outlined in Fig. 1. We summarize these special requirements as:

**Stand-alone**: The runtime verification framework should not only be deployed during the testing phase of the product but also after the product is shipped. Therefore, it should operate in a self-contained way and not depend on a powerful host computer that executes the observer.
**Non-intrusive**: The resulting observers should be efficient enough to not alter the timing requirements of the SUT. From an algorithmic viewpoint, observers with an a-priory known execution time are of utmost importance so as to statically determine upper bounds of the execution time of the observer. From an implementation point of view, we need to provide measures to passively observe events from the SUT.
**Timed**: To support correctness claims that involve timed properties, the framework should support expressive logics to formalize not only functional but also real-time requirements.
**Reconfigurable**: For the testing phase, the framework should be reconfigurable without requiring to re-synthesize the whole hardware design, which may take dozens of minutes to complete, for example when targeting an Field Programmable Gate Array (FPGA) platform.

## Contributions and roadmap

Our work can be seen as a response to overcome the above limitations that hinder the broad application of runtime verification to embedded real-time systems. This article provides the following contributions toward a *stand alone*, *non-intrusive*, *timed*, and *reconfigurable* hardware runtime verification approach:

- We present on-line observer algorithms that allow one to verify whether a past-time metric temporal logic (ptMTL) formula holds at (discrete) times $n \in\mathbb{N}_{0}$. The algorithms make use of basic operations only and are stated in a way that allows for a direct implementation in hardware, that can run without a host computer. By that our observers fulfill the timed and stand alone requirements.
- We formally prove the observers’ correctness and derive bounds on their time complexity in terms of gate delays and their space complexity in terms of required memory bits. With *n* being the time an observer algorithm is executed and *J* a non-empty interval we obtain, for the most general of the presented observer algorithms, the ptMTL Since operator *φ*_1_ *S*_*J*_ *φ*_2_, a time complexity of $\mathcal{O}(\log_{2}\log _{2}\max(J \cup\{n\}))$, only. The observer’s space complexity is dominated by the size of a list it needs to maintain. We show that the list’s space complexity is at most $2\lceil\log _{2}(n)\rceil\cdot(2\max(J)-\min(J)+2)/(2+\operatorname{len}(J))$, where $\operatorname{len}(J)=\max(J)-\min(J)$. Both complexity results, as well as the fact that our algorithms refrain from loops and recursions and build on simple operations only, enable applications of our runtime verification framework on resource limited platforms that require predictable timing and memory consumption.
- We explain how to derive non-instrumenting efficient realizations of the proposed observer algorithms in hardware. The resulting hardware profits from the simplicity and low complexity of our highly-parallel observer algorithms. In contrast to instrumentation-based runtime verification techniques for software systems our observers are well suited to supervise hardware components. By that, in combination with (b), our observers fulfill the non-intrusive requirement. Although our algorithms are tailored for a hardware implementation, the observers can simply be adopted to run in software too. Reconfigurability of our observers is achieved by, instead of hardwiring the observers inputs and outputs according to their parse tree, letting a programmable, specifically tailored microprocessor control a pool of observers.
- To evaluate the effectiveness of our approach, we report on a throughout study of simulation traces and synthesis results of a full-fledged hardware implementation of the presented observer algorithms and discuss the scalability of our approach.

With regard to the contributions above, (a) and (b) are an extension of our work we presented at the International Conference on Runtime Verification [71], including detailed correctness proofs for our algorithms and (c) and (d) are unique contributions of this article. Contribution (c) builds on our previous work [69], where we presented a microprocessor designed to evaluate ptLTL specifications in a software-oriented fashion. Using this approach to check ptMTL specifications, however, requires a costly (cf. Sect. 3.3) rewriting to an equivalent ptLTL specifications. Instead, we show how to map the building blocks of our ptMTL observer algorithms into efficient hardware units. This enables our microprocessor to natively evaluate ptMTL specifications in real-time. Both (c) and (d) help us to put the presented real-time observer algorithms into industrial practice.

The contributions of this article are presented as follows. First, Sect. 3 is a primer on temporal logics, which sets the scene for the monitoring algorithms stated in Sect. 4. Section 5 details the key structures of the hardware design and Sect. 6 reports on experimental evidence. We continue with a survey of related work in Sect. 7 and conclude in Sect. 8.

## Logics for runtime verification

We briefly summarize the temporal logics past-time linear temporal logic (ptLTL) and past-time metric temporal logic (ptMTL) which are used to specify properties in our framework. Both allow one to specify safety, past-time properties over executions. For further details, we refer the reader to more elaborate sources such as [2, 13, 32, 42, 51, 57].

### Past-time linear temporal logic

A popular logic in runtime verification is the past-time fragment of LTL (ptLTL), mainly due to: (i) observer generation for ptLTL is straightforward [39, Sect. 5], and (ii) ptLTL can easily express typical specifications [54]. Even though past-time operators do not yield the expressive power of full LTL [32, Sect. 2.6], past-time operators often express desired properties from specifications [50, 54]. With • in {∧,∨,→} and *σ* in the set *Σ* of atomic propositions, a formula *φ* is defined as: 
 Hereby, ⊙*φ* is the past-time analogue of next and referred to as *previously* *φ*. Likewise,  is referred to as *eventually in the past* *φ* and ⊡*φ* as *always in the past* *φ*. The duals of the until and the weak-until operators are *S*_*s*_ and *S*_*w*_, i.e., *strong since* and *weak since*, respectively. Similar as in LTL [41, Theorem 1], ptLTL can be reduced to the propositional operators plus two past-time operators [58], e.g., to ⊙ and *S*_*s*_. The satisfaction relation of a ptLTL specification can be defined as follows: Let *e*=(*s*_*t*_)_*t*≥0_ be an execution where *s*_*t*_ is a state of the system. Denote by *e* ^*n*^, for $n\in\mathbb{N}_{0}$, the *execution prefix* (*s*_*t*_)_0≤*t*≤*n*_. For a ptLTL formula *φ*, time $n\in\mathbb {N}_{0}$ and execution *e*, we define *φ* *holds at time n* *of execution* *e*, denoted *e* ^*n*^⊨*φ*, inductively as follows:

$$\begin{array}{llll} e^n \models true, & &\\ e^n \not\models\mathit{false}, & &\\ e^n \models\sigma,\ \text{where}\ \sigma\in\varSigma &~\textrm {iff}~& \sigma~\textrm{holds on}~s_n,\\ e^n \models\neg\varphi&~\textrm{iff}~& e^n \not\models\varphi,\\ e^n \models\varphi_1 \land\varphi_2 &~\textrm{iff}~& e^n \models \varphi_1 \ \text{ and } \ e^n \models\varphi_2,\\ e^n \models\varphi_1 \lor\varphi_2 &~\textrm{iff}~& e^n \models \varphi_1 \ \text{ or } \ e^n \models\varphi_2,\\ e^n \models\varphi_1 \to\varphi_2 &~\textrm{iff}~& e^n \models \varphi_1 \ \text{ implies } \ e^n \models\varphi_2,\\ e^n \models\odot\,\varphi&~\textrm{iff}~& e^{n-1} \models\varphi ~\textrm{if}~ n > 0~\text{, and }e^0\models\varphi\ \text{otherwise}, \\ e^n \models\varphi_1\,S_s\,\varphi_2 &~\textrm{iff}~& \exists j (0\le j \le n):~ (e^j \models\varphi_2~\land~\forall k (j < k \le n):~e^k \models\varphi_1 ).\\ \end{array} $$

The above syntax can be augmented with a set of additional operators [42, 51] to provide a succinct representation of common properties that appear in practice:

$$\begin{array}{lll} \varphi&::=& \uparrow\varphi~|~\downarrow\varphi~|~[\varphi ,\varphi)_{s}~|~[\varphi,\varphi)_{w} \end{array} $$

↑*φ* and ↓*φ* are trigger conditions where ↑*φ* stands for *start* *φ* (i.e., *φ* was false in the previous state and is true in the current state, equivalent to *φ*∧¬⊙*φ*), ↓*φ* for *end* *φ* (*φ* was true in the previous state and is false in the current state, equivalent to ¬*φ*∧⊙*φ*). The interval operators are strong *interval* [*φ*_1_,*φ*_2_)_*s*_ (*φ*_2_ was never true since the last time *φ*_1_ was true, including the state when *φ*_1_ was true, equivalent to ¬*φ*_2_∧((⊙¬*φ*_2_) *S*_*s*_ *φ*_1_)) and weak *interval* (equivalent to ⊡¬*φ*_2_∨[*φ*_1_,*φ*_2_)_*s*_) In the following we will only refer to the strong since and shortly write *S* instead of *S*_*s*_. Checking whether a ptLTL formula holds at time $n \in \mathbb{N}_{0}$ in some execution *e*=(*s*_*t*_)_*t*≥0_ can be determined by evaluating only the current state *s*_*n*_ and the results from the predecessor state *s*_*n*−1_ [42]. For example, evaluating the invariant *φ*=⊡ *σ* on execution *e*=(*s*_*t*_)_*t*≥0_ can be done by:

$$\begin{array}{lll} e^n \models\;\boxdot\sigma&\Leftrightarrow& \bigwedge_{t=0}^{n} (\sigma\text{ holds on } s_t )\\ &\Leftrightarrow& (e^{n-1} \models\boxdot\;\sigma ) \wedge ( \sigma\text{ holds on } s_n ) \end{array} $$

### Past-time metric temporal logic

MTL [2] extends LTL by replacing the qualitative temporal operators of LTL by quantitative operators that respect time bounds. Since we are interested in on-chip observer algorithms, progress of time is provided by the (possibly divided) chip’s clock signal, resulting in a discrete time base $\mathbb {N}_{0}$.^1^ Time bounds of quantitative operators are given in form of intervals: For *t* in $\mathbb{N}_{0}$ and *t*′ in $\mathbb{N}_{0}\cup\{\infty\}$, we write [*t*,*t*′) for the set $\{i \in \mathbb{N}_{0} \mid t\le i< t'\}$ and, if *t*′ in $\mathbb{N}_{0}$, [*t*,*t*′] for the set $\{ i \in \mathbb{N}_{0} \mid t\le i\le t'\}$. Similar to ptLTL, a restriction of MTL to its past time fragment (ptMTL) is of interest. Formally, a ptMTL formula *φ* is defined by:

$$\begin{array}{lll} \varphi&::=& \mathit{true}~\mid~\mathit{false}~\mid~\sigma~\mid~\neg\varphi~\mid~\varphi\bullet\varphi~\mid~\varphi~S_{J}~\varphi \end{array} $$

where *σ*∈*Σ*, •∈{∧,∨,→}, and *J*=[*t*,*t*′] for some $t,t' \in\mathbb{N}_{0}$. The semantics of *true*, *false*, *σ*, ¬*φ*, and *φ*•*φ* are as before. Recall that in ptLTL *φ*_1_ *S* *φ*_2_ expresses *φ*_2_ *was* true *in the past and since then* *φ*_1_ *was* true. By way of contrast, satisfaction of *e* ^*n*^⊨*φ*_1_ *S*_*J*_ *φ*_2_ in ptMTL, does not only depend on the observation that *φ*_1_ *S* *φ*_2_ holds in the current state, but also on (i) the time *n* of the current state and (ii) the times $i\in\mathbb{N}_{0}$ since when *φ*_1_ *S* *φ*_2_ was observed to be true: for at least one such *i*, *e* ^*i*^⊨*φ*_2_, and *n*−*i*∈*J* have to hold. Formally, we define:

$$\begin{array}{llllllll} e^n \models\varphi_{1}~S_J~\varphi_{2} & ~\textrm{iff}~& \exists i (0 \le i \le n): & \bigl(n-i \in J &\land& e^i \models\varphi _{2} &\land& \forall j(i< j \le n):~e^j \models\varphi_{1}\bigr) \end{array} $$

#### Example

Many real-time properties, such as

“*If the system leaves the idle mode, it has received an according signal in the past 50 clock-cycles.*”

can be expressed in ptMTL. The above property, e.g., can be formalized by:

$$\begin{aligned} \bigl( \downarrow\text{(in idle mode)} \bigr) \rightarrow \bigl( \mathit{true}~S_{[0,50]}~\text{(received message)} \bigr) \end{aligned}$$

Not surprisingly, determining satisfaction of an MTL (or ptMTL) formula is computationally more expensive than checking satisfaction of an LTL (or ptLTL) formula [78, Theorem 3.4].

### Rewriting past-time metric temporal logic to past-time linear temporal logic

In a discrete time setting, there is an equivalent ptLTL formula for every ptMTL formula [57], directly leading to an observer algorithm for *φ*_1_ *S*_[*a*,*b*]_ *φ*_2_. With ⊙^*i*^ *φ* being ⊙ applied *i* times to *φ*, a straightforward generic translation is given by the equivalence:

$$\begin{array}{llllllll} e^n \models\varphi_{1}~S_{[a,b]}~\varphi_{2} & ~\Leftrightarrow~ & \exists i (a \le i \le b): & ((\odot^i\varphi_{2}) \land(\odot ^{i-1}\varphi_1) \land(\odot^{i-2}\varphi_1) \land\dots\land \varphi_{1} ) \\ &~\Leftrightarrow~ & \bigvee_{i=a}^{b} & ( (\odot^i\varphi_2) \land\bigwedge_{j=0}^{i-1} (\odot^j\varphi_1) ) \end{array} $$

In a hardware implementation, one can make use of shift-registers to store the relevant part of the execution path with regard to the truth values of *φ*_1_ and *φ*_2_. We will proceed by a sample implementation making use of the equivalence above.

#### Example

**Fig. 2:**
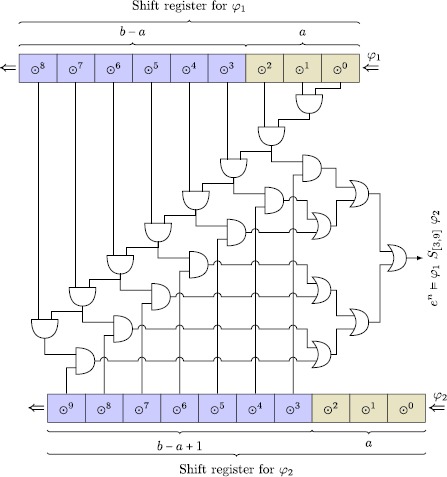
Hardware realization of a rewriting from *φ*_1_ *S*_[3,9]_ *φ*_2_ to $\bigvee_{i=a}^{b} ((\odot^{i}\psi) \land\bigwedge_{j=0}^{i-1} (\odot^{j}\varphi))$. The parameters *a* and *b* are set according to the interval in *φ*_1_ *S*_[3,9]_ *φ*_2_, i.e., *a*=3 and *b*=9, yielding $\bigvee_{i=3}^{9} ((\odot^{i}\psi) \land\bigwedge_{j=0}^{i-1} (\odot^{j}\varphi))$

Consider the ptMTL formula *φ*_1_ *S*_[3,9]_ *φ*_2_. Rewriting the formula into a hardware implementation, requires two shift registers of length 9 and 8, respectively. With the equivalences from above, *e* ^*n*^⊨*φ*_1_ *S*_[3,9]_ *φ*_2_ can be rewritten into $\bigvee_{i=3}^{9} ((\odot^{i}\varphi_{2}) \land \bigwedge_{j=0}^{i-1} (\odot^{j}\varphi_{1}))$, which can be realized by the optimized, hand-crafted circuit shown in Fig. 2. Observe that we do not need to store ⊙^0^ *φ*_1_ and ⊙^0^ *φ*_2_ explicitly, as they are immediately available. The circuit accounts for 15 two-input AND gates and six two-input OR gates. In a generalized setting, the proposed circuit requires the following resources: 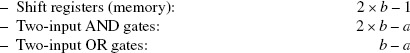
With parameters *a*=5 and *b*=1500, the circuit will occupy 3×*b*−2×*a*=3×1500−2×5=4490 two-input gates, and 2×*b*−1=2×1500−1=2999 flip-flops to implement the shift registers, resulting in a huge circuit.

It is important to observe that the chain of AND gates starting at ⊙^0^ *φ*_1_ introduces a gate propagation delay [44, Chap. 9] *Δ* on the signal that is proportional to *b* and delays the output of the verdict *e* ^*n*^⊨*φ*_1_ *S*_[*a*,*b*]_ *φ*_2_. With a propagation delay *δ*_AND_ of a single AND gate of and an AND chain of length *b*−1, the total propagation delay equals to *Δ*=(*b*−1)×*δ*_AND_. The chain becomes the *critical path* of the circuit and lowers the achievable operational frequency of the observer design. This effect can be alleviated by introducing a pipeline, however, not without the cost of additional memory and control logic.

This supports that rewriting ptMTL to ptLTL, albeit theoretically possible, is costly and thus infeasible in practice with an application in mind where the satisfaction relation is checked on-the-fly, i.e., in parallel to the SUT. Rewriting, however, may prove feasible when the observer is executed on a powerful host computer with a capable term rewriting engine at hand, as studied in [72].

## Observer design for real-time properties

In the following, we discuss the formal design of on-line observer algorithms for specifications in ptMTL in a discrete time model. The design is inspired by the observers described in [11] and extends work on observers for ptLTL [42] which have been built in hardware [63, 68]. We first give a high-level definition of the algorithms and turn to a hardware implementation in Sect. 5.

### Decomposing a specification

In the following let *e*=(*s*_*t*_)_*t*≥0_ be an execution and *φ* a ptMTL formula. Further, let *J*=[*t*,*t*′], with $t,t'\in\mathbb{N}_{0}$, be a non-empty interval. An observer is an algorithm that, given input *φ* and execution *e*, at each time $n\in\mathbb{N}_{0}$, returns true if *e* ^*n*^⊨*φ*, and false otherwise. We define the return value of our observer algorithm with input *φ* at time *n* by structural induction on ptMTL formula *φ*:

(i) *φ*=*true* returns true.
(ii) *φ*=*false* returns false.
(iii) *φ*=*σ*, where *σ*∈*Σ* returns true if *σ* holds on *s*_*n*_, and false otherwise.
(iv) *φ*=*φ*_1_•*φ*_2_ is true if *e* ^*n*^⊨*φ*_1_•*e* ^*n*^⊨*φ*_2_, where •∈{∧,∨,→}, and false otherwise.
(v) If *φ* is a ptLTL formula, we apply the algorithms proposed in [41, 42].
(vi) For *φ*=*φ*_1_ *S*_*J*_ *φ*_2_, we collect all times where *φ*_2_ was true in the past and since then *φ*_1_ remained true and store them in a list. At time *n* we check if there exists a time *τ* in the list such that *n*−*τ*∈*J*. If such a *τ* exists we return true, and false otherwise.

Algorithms for cases (i)–(iv) are straightforward. For case (v), we use the algorithm of Havelund and Roşu [41, 42], for which a translation into hardware building blocks (specified in terms of VHDL) is known [68]. Finding an efficient algorithm to detect satisfaction of *e* ^*n*^⊨*φ*_1_ *S*_*J*_ *φ*_2_ requires more sophisticated reasoning, and is the topic of the next sections. We start with efficient observer algorithms for the time-bounded variants of the ptLTL modalities ⊡*φ* and  and later extend them to an efficient observer algorithm for *φ*_1_ *S*_*J*_ *φ*_2_.

#### Running example

**Fig. 3:**
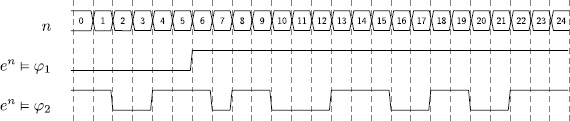
Validity of *e* ^*n*^⊨*φ*_1_ and *e* ^*n*^⊨*φ*_2_ for prefix of execution *e*

In the following, we frequently refer to the execution given in Fig. 3, which describes satisfaction of the two formulas *φ*_1_ and *φ*_2_ over times *n*∈[0,24]. We say *transition*  (*resp*. ) *of* *φ* *occurs at time* *n* iff *e* ^*n*^⊨ ↑*φ* in case *n*>0 and *e* ^0^⊨*φ* otherwise (resp. *e* ^*n*^⊨ ↓*φ* in case *n*>0 and *e* ^0^⊨¬*φ* otherwise). In the running example, transition  of *φ*_1_ occurs at time 6.

### The invariant and exists previously operators

We first discuss specializations of the common operators  (exists within interval *J*) and ⊡_*J*_ (invariant within interval *J*). In accordance with [6] we define both operators in terms of the Since operator by: 
1 From a practical point of view, two instances of the *exists within interval* and the *invariant within interval* operators, namely *invariant previously* () and *exists previously* (), where $\tau\in\mathbb {N}_{0}$, are valuable. They have the intended meaning *at least once in the past* *τ* *time units* () respectively *invariant for the past* *τ* *time units* (), and are defined by  respectively .

For example,  expresses that whenever *σ*_1_ becomes true, *σ*_2_ holds at all 10 previous time units. For both  and  we present simplifications that yield space- and time-efficient observers.

#### Invariant previously ()

is transformed into ¬(true *S*_[0,*τ*]_ ¬*φ*) by (1). An observer for  requires a single register  with domain $\mathbb{N}_{0} \cup\{ \infty \}$. Initially . Note that an actual implementation of this observer algorithm clearly must restrict itself to a bounded domain {0,1,…,*N*}∪{∞}, where *N* is chosen sufficiently large to cover the expected mission time of the system being analyzed. We will discuss implementation considerations of our observers in Sect. 5 and meanwhile assume unbounded domain registers.

For the observer in Algorithm 1, we define predicate  as: 
 Intuitively, the predicate  holds, and thus the algorithm returns true at time *n*, iff the latest  transition of *φ* occurred before *n*−*τ* and no  transition of *φ* occurred since then until time *n*.

**Algorithm 1:**
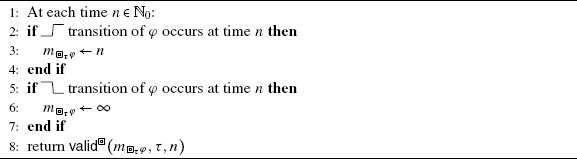
Observer for . Initially, .

#### Theorem 1

*For all* $n\in\mathbb{N}_{0}$, *the observer stated in Algorithm* 1 *implements* .

#### Proof

We first observe the equivalences 
2 Note that interval [max(0,*n*−*τ*),*n*] is never empty. Thus equation (2) holds iff a  transition of *φ* occurred at a time at most max(0,*n*−*τ*) and no  transition of *φ* occurred since then until time *n*. The theorem follows. □

#### Running example

Consider  on the execution in Fig. 3. Initially, . At time 0, *φ*_2_ holds and thus . The predicate  holds, the algorithm returns true and we have that . For similar arguments, at time 1, . At time 2, a  transition of *φ*_2_ occurs and we have . Since predicate  does not hold, we have that . For similar arguments, at time 3, . Since a  transition of *φ*_2_ occurs at time 4, . Again,  does not hold, thus, . The same is true for time 5, thus, . At time 6, ↑*φ*_1_ becomes true and since  is true, we deduce *e* ^6^⊨*ψ*. For times *n*′ prior to 6, (i.e., 0≤*n*′<6), the left-hand side of the implication of *ψ* does not hold. We immediately have that *e* ^*n*′^⊨*ψ*.

#### Exists previously ()

From the equivalence , we can immediately derive an observer for  from the observer for . The resulting algorithm can straightforwardly be implemented by checking for a  (resp. ) transition of *φ* instead of a  (resp. ) transition of ¬*φ* in line 2 (resp. line 5) and negating the output in line 8.

### The invariant and exists within interval operators

We now present observers for the more general operators *invariant within interval* *J* (⊡_*J*_) and *exists within interval* *J* (). Instead of a register (such as  in case of the observer for ), both observers require a list of time point pairs. Clearly, an efficient implementation of this list is vital for an efficient observer. In the following, we present several techniques so as to keep the list succinct, whilst preserving validity of the observer. For a list *l*, we denote by |*l*| its length, and by *l*[*k*], where $k\in\mathbb{N}$, its *k*th element. We assume that elements are always appended to the tail of a list.

#### Invariant within interval (⊡_*J*_ *φ*)

is transformed into ¬(true *S*_*J*_ ¬*φ*) by (1). An observer for ⊡_*J*_ *φ* requires a list $l_{\boxdot_{J}\varphi}$ of elements from $(\mathbb{N}_{0} \cup\{ \infty\} )^{2}$. For a pair of time points $T\in(\mathbb{N}_{0} \cup\{ \infty\} )^{2}$, we shortly write *T*.*τ*_*s*_ for its first component and *T*.*τ*_*e*_ for its second component. Initially, $l_{\boxdot_{J}\varphi}$ is empty. For the observer in Algorithm 2, we define predicate valid ^⊡^(*T*,*n*,*J*), with $T\in(\mathbb{N}_{0} \cup\{ \infty\} )^{2}$, by:

$$\mathsf{valid}^{\boxdot}(T,n,J) \equiv\bigl(T.\tau_s \leq\max \bigl(0,n-\max (J)\bigr)\bigr) \land\bigl(T.\tau_e \ge n - \min(J) \bigr), $$

and predicate feasible(*T*,*n*,*J*) as:

$$\mathsf{feasible}(T,n,J) \equiv\bigl(T.\tau_e - T. \tau_s \ge \operatorname{len}(J)\bigr) \vee\bigl(T. \tau_s = 0 \, \wedge\, T.\tau_e \ge n-\min(J)\bigr). $$

Intuitively, Algorithm 2 keeps track of all maximal intervals where *φ* holds whose length is large enough to potentially lead to the satisfaction of ⊡_*J*_ *φ*. Whether this is the case is determined by the fact whether a tuple representation of an interval satisfies the feasible predicate. For large *n*, this means that an interval has to have length at least $\operatorname{len}(J)$.

**Algorithm 2:**
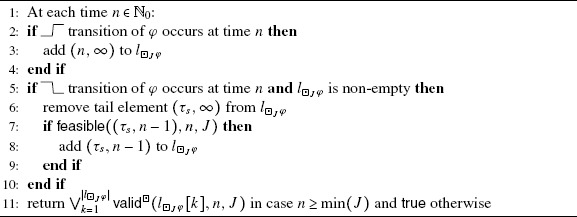
Observer for ⊡_*J*_ *φ*. Initially, $l_{\boxdot _{J}\varphi} = ()$.

We will deduce the correctness of the observer stated in Algorithm 2 from the correctness of a generalized algorithm, presented in Sect. 4.4, obtaining:

#### Theorem 2

*For all* $n\in\mathbb{N}_{0}$, *the observer stated in Algorithm* 2 *implements* *e* ^*n*^⊨⊡_*J*_ *φ*.

#### Running example

Consider *ψ*≡(↑*φ*_1_)→(⊡_[3,4]_ *φ*_2_) and execution *e* of Fig. 3. At time 0, the element (0,∞) is inserted into $l_{\boxdot_{[3,4]} \varphi_{2}}$. The  transition of *φ*_2_ at time 2 then leads to $l_{\boxdot_{[3,4]} \varphi_{2}} = ((0, 1))$, since feasible((0,1),2,[3,4]) holds. At time 4, another pair is added, resulting in $l_{\boxdot_{[3,4]} \varphi_{2}} = ((0,1),(4,\infty))$. Since at time 6:

$$\begin{array}{lllll} \mathsf{valid}^{\boxdot}\bigl(l_{\boxdot_{[3,4]}\varphi_2}[1],6,[3,4]\bigr) & \Leftrightarrow& (0 \leq6 - 4) \land(1 \ge6 - 3) & \Leftrightarrow & \mathsf{false} \\ \mathsf{valid}^{\boxdot}\bigl(l_{\boxdot_{[3,4]}\varphi_2}[2],6,[3,4]\bigr) & \Leftrightarrow& (4 \leq6 - 4) \land(\infty\ge6 - 3) & \Leftrightarrow& \mathsf{false} \end{array} $$

we obtain $e^{6} \not\models\psi$.

#### Exists within interval ()

From the equivalence , we can easily derive an observer for  from the observer for ⊡_*J*_ *φ*. As before, we obtain the observer by swapping  and  transitions and negating the output.

### The since within interval operator

An observer for *φ*_1_ *S*_*J*_ *φ*_2_ is obtained from a  observer and additional logic to reset the observer’s list. Let *l*_*S*_ be an initially empty list. The *φ*_1_ *S*_*J*_ *φ*_2_ observer is stated in Algorithm 3. In case *φ*_1_ holds at time *n*, the observer executes the same code as a  observer. In case *φ*_1_ does not hold at time *n*, the list $l_{\varphi_{1} S_{J} \varphi_{2}}$ is reset to contain only a single entry whose content depends on the validity of *φ*_2_. Intuitively, for the maximum suffix where *φ*_1_ holds Algorithm 3 keeps track of all maximal intervals where *φ*_2_ holds whose length is large enough to potentially lead to the satisfaction of *φ*_1_ *S*_*J*_ *φ*_2_.

**Algorithm 3:**
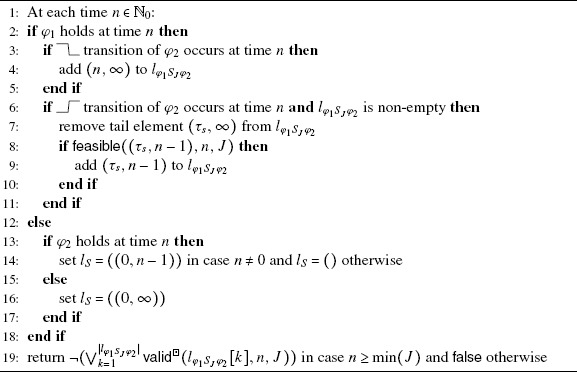
Observer for *φ*_1_ *S*_*J*_ *φ*_2_. Initially, $l_{\varphi_{1} S_{J} \varphi_{2}} = ()$.

#### Theorem 3

*For all* $n\in\mathbb{N}_{0}$, *the observer in Algorithm* 3 *implements* *e* ^*n*^⊨*φ*_1_ *S*_*J*_ *φ*_2_.

For the proof we introduce additional notation. For list *l* denote with *l*⋅*T*, the list resulting from adding element *T* to the tail of list *l*. Further denote with *l* ^*n*^, where $n\in\mathbb{N}_{0}$, the state of Algorithm 3’s list *l*_*S*_ in line 19 executed at time *n*. By $\overline{l}^{n}$ we denote the set [0,*n*]∖⋃_1≤*k*≤|*l*|_[*l*[*k*].*τ*_*s*_,*l*[*k*].*τ*_*e*_+1). For example, if *l* ^10^=((0,3),(5,8)), then $\overline{l}^{10} = \{4,9,10\}$. We first show that the following proposition holds:

#### Proposition 1

*Consider Algorithm* 3 *without the feasibility check in line *8, *i*.*e*., *replace this line with “if* true *then”*. *For the modified algorithm the following is correct*: *For all* $n\in\mathbb{N}_{0}$ *and* *i*≤*n*, $i\in\overline{l}^{n}$ *holds iff both* *e* ^*i*^⊨*φ*_2_ *and for all* *k*,*i*<*k*≤*n*, *e* ^*k*^⊨*φ*_1_.

#### Proof

The proof is by induction on $n\in\mathbb{N}_{0}$.

**Begin** (*n*=0): Consider the four cases for *φ*_1_ and *φ*_2_:

*Case (i):* Assume *e* ^*n*^⊨*φ*_1_ and $e^{n} \not\models \varphi_{2}$. Then *l* ^*n*^=((0,∞)) and thus $\overline{l}^{n} = \emptyset$. Since $e^{n} \not\models\varphi_{2}$, the induction basis follows in this case.

*Case (ii):* Assume *e* ^*n*^⊨*φ*_1_ and *e* ^*n*^⊨*φ*_2_. Then *l* ^*n*^=() and thus $\overline{l}^{n} = \{0\}$. Since *e* ^*n*^⊨*φ*_2_, the induction basis follows in this case.

*Case (iii):* Assume $e^{n} \not\models\varphi_{1}$ and $e^{n} \not \models \varphi_{2}$. The arguments are analogous to the arguments of case (i).

*Case (iv):* Assume $e^{n} \not\models\varphi_{1}$ and *e* ^*n*^⊨*φ*_2_. The arguments are analogous to the arguments of case (ii).

**Step** (*n*−1→*n*): Assume that the statement holds for *n*−1≥0. We will show that it holds for *n*, too. Thereby we consider the same cases (i) to (iv) as in the induction basis.

*Case (i):* We distinguish two cases for *φ*_2_: a  transition of *φ*_2_ (i.a) did, or (i.b) did not occur at time *n*.

In case of (i.a), *l* ^*n*^=*l* ^*n*−1^⋅(*n*,∞). Thus $\overline{l}^{n} = \overline{l}^{n-1}$. Since *e* ^*n*^⊨*φ*_1_ but $e^{n} \not\models\varphi_{2}$, the induction step follows in this case.

In case of (i.b), *l* ^*n*^=*l* ^*n*−1^. By the algorithm, the last element in *l* ^*n*^ must be of the form (*n*′,∞) with *n*′<*n*. Thus $\overline{l}^{n} = \overline{l}^{n-1}$. Again, the induction step follows in this case.

*Case (ii):* We distinguish two cases for *φ*_2_: a  transition of *φ*_2_ (ii.a) did, or (ii.b) did not occur at time *n*.

Now consider case (ii.a): If *l* ^*n*−1^=(), *l* ^*n*^=*l* ^*n*−1^ holds, and thus $\overline{l}^{n}=\overline{l}^{n-1}\cup\{n\}$. Otherwise, the last element in *l* ^*n*−1^, say (*n*′,∞), with *n*′≤*n*, is replaced with (*n*′,*n*) in *l* ^*n*^. Again, $\overline{l}^{n}=\overline{l}^{n-1}\cup\{n\}$. In both cases, the induction step follows, as *e* ^*n*^⊨*φ*_1_ and *e* ^*n*^⊨*φ*_2_.

In case of (ii.b), *l* ^*n*^=*l* ^*n*−1^. By the algorithm, the last element in *l* ^*n*^, if it exists, must be of the form (*n*′,*n*″) with *n*′≤*n*″<*n*. Thus $\overline{l}^{n} = \overline{l}^{n-1}\cup\{n\}$. Again, the induction step follows in this case.

*Case (iii):* By the algorithm, *l* ^*n*^=((0,∞)). Thus $\overline{l}^{n} = \emptyset$. Since $e^{n} \not\models \varphi_{2}$, the induction step follows in this case.

*Case (iv):* By the algorithm, and since *n*>0, *l* ^*n*^=((0,*n*−1)). Thus $\overline{l}^{n} = \{n\}$. Since *e* ^*n*^⊨*φ*_1_, the induction step follows in this case. □

We are now in the position to prove Theorem 3.

#### Proof of Theorem 3

Consider the modified Algorithm 3 without feasibility check. By analogous arguments as in the proof of Theorem 1, we obtain

$$\begin{aligned} &e^n \models\varphi_1\,S_{J}\, \varphi_2 \\ &\Leftrightarrow\forall i: i\in[0,n] \cap\bigl[n-\max(J),n-\min(J)\bigr] \wedge \bigl(e^i \models\varphi_2\bigr) \wedge\forall k(i<k\le n): e^k \models \varphi_1 \\ &\Leftrightarrow\forall i: i\in\bigl[\max\bigl(0,n-\max(J)\bigr),n-\min(J)\bigr] \wedge\bigl(e^i \models\varphi_2\bigr) \wedge\forall k(i<k\le n): e^k \models\varphi _1 . \end{aligned}$$

We distinguish two cases for *n*, namely (i) *n*<min(*J*), and (ii) *n*≥min(*J*).

(i) In case *n*<min(*J*), interval [max(0,*n*−max(*J*)),*n*−min(*J*)] is empty, and *e* ^*n*^⊨*φ*_1_ *S*_*J*_ *φ*_2_ is trivially false. Since the algorithm returns false in this case, the theorem follows for Algorithm 3 without the feasibility check for case (i).

(ii) In case *n*≥min(*J*), interval *I*=[max(0,*n*−max(*J*)),*n*−min(*J*)] is non-empty. Thus *e* ^*n*^⊨*φ*_1_ *S*_*J*_ *φ*_2_ holds iff there exists an *i*∈*I* for which *e* ^*i*^⊨*φ*_2_ and for all *k*,*i*<*k*≤*n*, *e* ^*k*^⊨*φ*_1_. From Proposition 1 we know that this is the case iff there exists an *i*∈*I* with $i\in\overline{l}^{n}$. The latter is the case iff there exists no tuple (*τ*_*s*_,*τ*_*e*_) in *l* ^*n*^ with valid ^⊡^((*τ*_*s*_,*τ*_*e*_),*n*,*J*). Since, for *n*≥min(*J*), the algorithm returns true iff this is the case, the theorem follows for Algorithm 3 without the feasibility check for case (ii).

It remains to show that the theorem holds for Algorithm 3 with original line 8. If we can show that from ¬feasible((*τ*_*s*_,*τ*_*e*_),*n*,*J*) follows ¬valid ^⊡^((*τ*_*s*_,*τ*_*e*_),*n*′,*J*), for all times *n*′≥*n*, we may safely remove tuple (*τ*_*s*_,*τ*_*e*_) from the algorithm’s list without changing the algorithm’s return value.

Assume that valid ^⊡^((*τ*_*s*_,*τ*_*e*_),*n*′,*J*) holds, with *n*′≥*n*. We distinguish two cases for *n*′: (a) *n*′<max(*J*) and (b) *n*′≥max(*J*):

(a) In case *n*′<max(*J*), it follows from valid ^⊡^((*τ*_*s*_,*τ*_*e*_),*n*′,*J*) that *T*.*τ*_*s*_=0 and *T*.*τ*_*e*_≥*n*′−min(*J*)≥*n*−min(*J*). Thus feasible((*τ*_*s*_,*τ*_*e*_),*n*,*J*) holds.

(b) Otherwise *n*′≥max(*J*), and it follows from valid ^⊡^((*τ*_*s*_,*τ*_*e*_),*n*′,*J*) that *T*.*τ*_*s*_≤*n*′−max(*J*) and *T*.*τ*_*e*_≥*n*′−min(*J*). Thus $T.\tau_{e}-T.\tau_{s} \le\operatorname{len}(J)$ and thereby feasible((*τ*_*s*_,*τ*_*e*_),*n*,*J*).

The theorem follows. □

With the two definitions in (1), an observer algorithm implementing *e* ^*n*^⊨⊡_*J*_ *φ* can be deduced from Algorithm 3 by negating its input, its output, and replacing the if condition in line 2 by true. Since the obtained algorithm is equivalent to Algorithm 2, Theorem 2 immediately follows.

### Garbage collection

Thus far, we did not consider housekeeping of either list so as to control the growth of the lists. It is important to appreciate that each timed operator has a bounded time-horizon on which it depends. This horizon can be exploited to eliminate pairs *T* from Algorithm 2 or Algorithm 3’s lists that can neither validate nor invalidate the specification. Our garbage collector works as follows: at any time $n\in\mathbb {N}_{0}$, we remove a tuple *T* from the list if the proposition

$$\mathsf{garbage}(T, n, J) \equiv T.\tau_e < n - \min(J) $$

holds. The main purpose of the garbage collector is to reduce the algorithms’ space and time complexity: We will show that, by removing tuples, garbage collection considerably reduces the algorithms’ space complexity. Further, observe that direct implementations of line 11 of Algorithm 2 and line 19 of Algorithm 3 require searches through a list. We will show that, with our garbage collector running in parallel to the observer algorithms, these lines reduce to checking the list’s first element only. Thus we may replace the list in both algorithms by a simple queue, where elements are added only to its tail and read and removed only at its head.

In the following, we show the correctness of our garbage collection strategy for any of the proposed algorithms: We first show that if a tuple *T* is allowed to be removed by the garbage collector at time *n*, it cannot satisfy valid ^⊡^ at that time or at any later time. It is thus safe to remove it from the list.

#### Lemma 1

*If* garbage(*T*,*n*,*J*), *then* ¬valid ^⊡^(*T*,*n*′,*J*) *for all* *n*≥*n*′.

#### Proof

Assume that garbage(*T*,*n*,*J*) holds. Then *T*.*τ*_*e*_<*n*−min(*J*)≤*n*′−min(*J*). Since *T*.*τ*_*e*_≥*n*′−min(*J*) is necessary for valid ^⊡^(*T*,*n*′,*J*) to hold, the lemma follows. □

We next show that always a prefix of a list is removed. This allows the garbage collector to evaluate garbage iteratively, starting from the head of the list.

For that purpose we introduce additional notation. We write “…” for a potentially empty sequence of tuples. For example, (…,*T*,*T*′,…) denotes a list of length at least two, where *T* and *T*′ are any two successive elements in this list.

#### Lemma 2

*Let* *l*=(…,*T*,*T*′,…) *be the list of any of the proposed observer algorithms at time* $n\in\mathbb{N}_{0}$. *If* garbage(*T*′,*n*,*J*), *then* garbage(*T*,*n*,*J*).

#### Proof

Assume that garbage(*T*′,*n*,*J*) holds. Then *T*′.*τ*_*e*_<*n*−min(*J*). By observing that all of the proposed algorithms ensure that *T*.*τ*_*e*_≤*T*′.*τ*_*e*_ for successive list elements *T* and *T*′, we obtain *T*.*τ*_*e*_<*n*−min(*J*), i.e., garbage(*T*,*n*,*J*) holds. The lemma follows. □

We next prove an upper bound on the length of Algorithm 2 or Algorithm 3’s lists. We start by showing that there is a minimum distance between successive elements in the algorithms’ lists.

#### Lemma 3

*Let* *l*=(…,*T*,*T*′,…) *be the list of any of the proposed observer algorithms at time* $n\in\mathbb{N}_{0}$. *Then* *T*.*τ*_*e*_+2≤*T*′.*τ*_*s*_.

#### Proof

Consider Algorithm 2. By the algorithm, tuple *T* must have been added by line 8. For line 8 to add *T*=(*T*.*τ*_*s*_,*n*−1), transition  of *φ* must have occurred at time *n*. Thus the next tuple added to the list at a time *n*′>*n* must have been of the form (*n*′,∞). Since, by the algorithm, then *T*′.*τ*_*s*_≥*n*′ must hold, we further obtain *T*′.*τ*_*s*_≥(*n*−1)+2=*T*.*τ*_*e*_+2. The lemma follows for Algorithm 2.

For Algorithm 3 the lemma follows by analogous arguments. □

Further the first element in the list that was not removed by the garbage collector cannot be of arbitrary age:

#### Lemma 4

*Consider a time*-*bounded formula* ⊡_*J*_ *φ*, , *or* *φ*_1_ *S*_*J*_ *φ*_2_. *Let* *l*=(*T*,…) *be the list of the proposed respective observer algorithm at time* $n\in\mathbb{N}_{0}$, *after garbage collection has run at time n*. *Then* *T*.*τ*_*e*_≥*n*−min(*J*).

#### Proof

It must hold that garbage(*T*,*n*,*J*) is false, since otherwise *T* would have been removed by the garbage collector. Thus *T*.*τ*_*e*_≥*n*−min(*J*). □

#### Lemma 5

*Let* *l* *be the list of any of the proposed observer algorithms at time* $n\in\mathbb{N}_{0}$, *after garbage collection has run at time n*, *and assume that* *l* *is non*-*empty*. *Let* *T* ^*k*^=*ℓ*[*k*], *for* 1≤*k*≤|*ℓ*|. *Then* $T^{k}.\tau_{e}\ge n-\min(J)+(k-1)(2+\operatorname{len}(J))$.

#### Proof

The proof is by induction on the number *k*≥1 of the element in the list.

**Begin** (*k*=1): Immediately follows from Lemma 4.

**Step** (*k*−1→*k*): Assume that the statement holds for *k*−1≥1. We will show that it holds for *k*, too. By Lemma 3,

$$\begin{aligned} T^k.\tau_s\ge T^{k-1}.\tau_e +2. \end{aligned}$$

Because *k*>1, it must hold that *T* ^*k*^.*τ*_*s*_≠0. Thus, by the algorithms, either feasible(*T* ^*k*^,*n*′,*J*) must have held at time *n*′≤*n*, when *T* ^*k*^ was added to the list, or *T* ^*k*^=(*n*′,∞). In both cases,

$$\begin{aligned} T^k.\tau_e\ge T^k.\tau_s + \operatorname{len}(J). \end{aligned}$$

It follows that,

3 $$\begin{aligned} T^k.\tau_e\ge T^{k-1}.\tau_e +2+ \operatorname{len}(J). \end{aligned}$$

Combining (3) and the induction hypothesis

$$\begin{aligned} T^{k-1}.\tau_e \ge n-\min(J) + (k-2) \bigl(2+ \operatorname{len}(J)\bigr) \end{aligned}$$

thus yields,

$$\begin{aligned} T^k.\tau_e \ge n-\min(J) + (k-1) \bigl(2+ \operatorname{len}(J)\bigr). \end{aligned}$$

The lemma follows. □

We may now derive an upper bound on the number of list elements for all our observer algorithms:

#### Theorem 4

*Consider a time*-*bounded formula* ⊡_*J*_ *φ*, , *or* *φ*_1_ *S*_*J*_ *φ*_2_. *Let* *l* *be the list of the proposed respective observer algorithm at time* $n\in\mathbb{N}_{0}$, *after garbage collection has run at time n*. *Then* *l* *is of length at most*

$$ \frac{2\max(J)-\min(J)+2}{2+\operatorname{len}(J)}. $$

#### Proof

In case *l* is empty the lemma follows trivially. Assume *l*=(*T* ^1^,…,*T* ^*k*^) is non-empty. We distinguish two cases for *T* ^*k*^:

(i) In case *T* ^*k*^.*τ*_*e*_≠∞, we obtain from Lemma 5,

4 $$\begin{aligned} &T^k.\tau_e \ge n-\min(J) + (k-1) \bigl(2+ \operatorname{len}(J)\bigr) . \end{aligned}$$

Further, by the algorithms, a finite *T* ^*k*^.*τ*_*e*_ implies that

5 $$\begin{aligned} T^k.\tau_e \le n-1. \end{aligned}$$

Combination of (4) and (5) yields

$$\begin{aligned} n-1 &\ge n-\min(J) + (k-1) \bigl(2+\operatorname{len}(J)\bigr)\ \Leftrightarrow \\ k &\le\frac{\max(J)+1}{2+\operatorname{len}(J)} \le\frac{2\max(J)-\min(J)+2}{2+\operatorname{len}(J)} . \end{aligned}$$

The theorem follows for this case.

(ii) Otherwise, i.e., in case *T* ^*k*^.*τ*_*e*_=∞, by the algorithms,

6 $$ T^k.\tau_s \le n $$

must hold. We obtain from Lemma 5,

$$\begin{aligned} T^{k-1}.\tau_e \ge n-\min(J) + (k-2) \bigl(2+\operatorname {len}(J)\bigr), \end{aligned}$$

and by Lemma 3,

7 $$\begin{aligned} T^{k}.\tau_s \ge n-\min(J) +2 + (k-2) \bigl(2+ \operatorname {len}(J)\bigr). \end{aligned}$$

Combination of (6) and (7) yields

$$\begin{aligned} n &\ge n-\min(J) +2 +(k-2) \bigl(2+\operatorname{len}(J)\bigr)\ \Leftrightarrow \\ k &\le\frac{2\max(J)-\min(J)+2}{2+\operatorname{len}(J)} . \end{aligned}$$

The theorem also follows for this case. □

### Discussion of space and time complexity

We first give a bound on space complexity in terms of single-bit registers that are required by a hardware implementation of our observer algorithms. Clearly, the space complexity for an observer of ptMTL formula *φ* is the sum of the space complexity of its observers for all subformulas of *φ*, and its time complexity scales with the depth of the parse tree of *φ*. It is thus sufficient to state bounds for ⊡_*J*_ *φ*, , and *φ*_1_ *S*_*J*_ *φ*_2_. In all these cases the respective observer algorithm’s space complexity is dominated by the space complexity of the algorithm’s list. Clearly the bit complexity of the *τ*_*s*_ or *τ*_*e*_ component of a tuple added by one of the proposed algorithms to its list before time $n\in\mathbb{N}_{0}$ is bounded by ⌈log_2_(*n*)⌉. We thus obtain from Theorem 4 that for any of the time-bounded formulas ⊡_*J*_ *φ*, , or *φ*_1_ *S*_*J*_ *φ*_2_, our proposed observer algorithms, if executed at time $n\in\mathbb{N}_{0}$, have to maintain a list of space complexity at most:

8 $$ 2\bigl\lceil \log_2(n)\bigr\rceil \cdot\frac{2\max(J)-\min (J)+2}{2+\operatorname{len}(J)}. $$

**Fig. 4:**
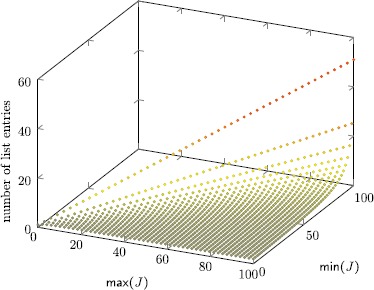
Visualization of the space complexity bound $(2\cdot\max (J)-\min(J)+2)/ (2+\operatorname{len}(J))$ for 0≤min(*J*)≤max(*J*)≤100 with 50 samples per axis

Figure 4 visualizes this bound, revealing that memory consumption is moderate for almost all cases, except for configurations where min(*J*)=max(*J*), where space complexity grows linear in max(*J*). Note that log_2_(*n*) is small for realistic experimental setups. For example, allowing to store 52 bit per tuple component is sufficient to check executions that are sampled with a 1 MHz clock during a period of over 140 years.

An alternative to storing absolute times in the observer’s list, is to adapt the observer algorithms in a way such that only relative times are stored. While this potentially reduces the bound of Eq. (8) by substituting log_2_(*n*) with log_2_(max(*J*)), it requires updating of the list elements (as these then contain relative times) at every time $n\in\mathbb{N}_{0}$. Since this would require more complex hardware mechanism and result in a slower on-line algorithm, we decided not to follow this path in our hardware implementation.

We next show that garbage collection allows one to reduce time complexity of the proposed observers. The time-determining part of Algorithms 2 and 3 is the evaluation of the predicate valid ^⊡^ for all list elements in line 11 and line 19 respectively. However, garbage collection makes it possible to only evaluate the predicate for the first element in the list, thus greatly improving time complexity of the proposed algorithms:

#### Lemma 6

*Let* *l*=(*T*,…,*T*′,…) *be the list of any of the observer algorithms at time* $n\in\mathbb{N}_{0}$, *after garbage collection has run at time n*. *Then* ¬valid ^⊡^(*T*′,*n*,*J*).

#### Proof

Assume by means of contradiction that valid ^⊡^(*T*′,*n*,*J*) holds. Then *T*′.*τ*_*s*_≤max(0,*n*−max(*J*))≤max(0,*n*−min(*J*)). For both Algorithms 2 and 3 we observe that *T*.*τ*_*e*_<*T*′.*τ*_*s*_ has to hold. Thus *T*.*τ*_*e*_<max(0,*n*−min(*J*)). Since neither Algorithms 2 nor 3 add tuples with a negative *τ*_*s*_ or *τ*_*e*_ component, we obtain that *T*.*τ*_*e*_<*n*−min(*J*) has to hold and by that garbage(*T*,*n*,*J*) holds. A contradiction to the fact that garbage collection has been run at time *n*: it would have removed tuple *T* in that case. The lemma follows. □

Since further there exist circuits that perform an addition of two integers of bit complexity $w \in\mathbb{N}$ within time $\mathcal{O}(\log_{2}(w))$ [47], and since evaluating the valid ^⊡^(*T*,*n*,*J*) and garbage(*T*,*n*,*J*) predicates at time $n\in\mathbb{N}_{0}$ requires addition of integers of bit complexity at most max(log_2_(*n*),log_2_(*J*)), we arrive at an asymptotic time complexity of

$$ \mathcal{O} \bigl(\log_2\log_2\max\bigl(J\cup\{n\}\bigr) \bigr), $$

for any of the observers ⊡_*J*_ *φ*, , and *φ*_1_ *S*_*J*_ *φ*_2_ executed at time *n*.

## Mapping the framework into hardware structures

**Fig. 5:**
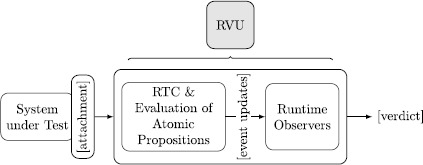
The runtime verification unit (RVU) and its architecture

In what follows, we elaborate design considerations to map the proposed runtime verification framework into hardware. Figure 5 shows the main modules of a hardware instance of the framework, i.e., the runtime verification unit (RVU). The design of the RVU is generic and can be attached to various SUTs, as shown in Fig. 1. We start with a discussion of how our RVU connects to existing systems and how we map registers and lists into primitive hardware structures. We then show how we derive the current time from a Real-Time Clock (RTC) and how we evaluate atomic propositions, before we show how to adapt an existing low-footprint, programmable ptLTL verification microprocessor to also evaluate ptMTL specifications using the observer algorithms described in Sect. 4.

### Interfacing the system under test

Our runtime verification unit (see Fig. 5) connects to various systems through wiretapping of the SUT’s communication interfaces, as outlined in Fig. 1. The attachment to these communication interfaces is application specific. In its current shape, we implemented bus interfaces for systems operating with: RS-232 (serial port), CAN (vehicle bus), Wishbone (System-on-Chip interconnect), I^2^C (multimaster serial bus), and JTAG (boundary scan) variants.

### Registers and lists of pairs of time points

Registers are implemented by, for example, linking multiple flip-flops. The width of such a register equals to the width of the (upper bounded) time points issued by the RTC plus two additional bits. These additional bits enable indication of overflows when performing arithmetics on time points and indication of the special value ∞. For lists of pairs of time points, we turn to block RAMs, which we organize as ring buffers. Each ring buffer is managed by a unit that controls its read pointer (RP) and its write pointer (WP).

### Real-time clock

The progression of time is measured by a digital clock, i.e., the real-time-clock (RTC), which contains a counter and an oscillation mechanism that periodically increments the counter [48, Chap. 3]. For an on-chip RVU solution, the oscillation mechanism can also be bounded to the global system clock of the SUT. Note that the design also allows for an instantiation of a fully external clock which is decoupled from the SUT, such as a GPS receiver. Time points are internally stored in registers of width *w*=⌈log_2_(*N*)⌉+2, where *N* is the maximum time (in terms of ticks of the RTC) expected to occur during a run of the SUT. The two additional bits enable indication of overflows when performing arithmetical operations on time points and indication of ∞.

Note that our proposed algorithms (cf. Sect. 4) make use of absolute time points, i.e., we store time points for both  and  transitions of an event *e*. In contrary, we could also use a mixed representation of absolute and relative time points, i.e., store the absolute time points of the  transition of event *e* and then count the duration of *e* (the number of clock ticks until the  transition occurs). While the latter would help to improve the average-case memory requirements in a software-oriented implementation, the former is superior in terms of a hardware implementation: In a hardware design, memory needs to be statically assigned at design time; thus registers have to be of width *w* rendering the benefits of relative time points. Further storing relative time points would require an additional counter of width *w* for all atomic propositions and subformulas that use time points.

### Evaluation of atomic propositions

Ideally, with respect to expressiveness of the supported specifications, atomic propositions include arbitrary equalities, inequalities, and disequalities over variables in the state of the SUT. To arrive at a responsive framework, however, an observer needs to guarantee that it finishes evaluation of atomic propositions within a tight time bound. It is therefore necessary to establish a balance between (hardware) complexity of the resulting observer and expressiveness. To achieve this balance, we restrict the class of atomic propositions supported by our framework in a way inspired by the so-called logahedron abstract domain [45], frequently used in the field of abstract interpretation [24].

Specifically, the class of supported atomic propositions consist of conjunctions of linear constraints, where each constraint ranges over two variables. In addition, each variable can be negated and multiplied by a power of two. In our implementation, we support atomic propositions that are restricted linear constraints ranging over values transferred through an interface of the SUT. Specifically, atomic propositions are of the form (±2^*n*^⋅*v*_1_±2^*m*^⋅*v*_2_)⋈*c*, where *v*_1_ and *v*_2_ are application specific symbols, $c,n,m \in\mathbb{Z}$ and ⋈∈{=,≠,≤,≥,>,<}. For example, when the RVU is connected to a microcontroller data bus (cf. Fig. 1), *v*_1_ (and *v*_2_) can be interpreted as the value stored in a memory location, which in turn, maps to a program variable.

**Fig. 6:**
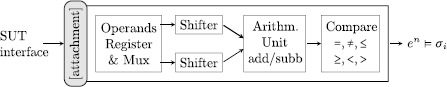
An AtChecker unit to evaluate an atomic proposition *σ*_*i*_ in hardware

In [68, Sect. 3] we showed how to build circuits (see Fig. 6) that evaluate such linear constraints, with a minimum time penalty. We will use the term AtChecker to refer to such a circuit. It comprises an operands register to fetch new data from the SUT interface, two shifter units to implement multiplication and division by a power of two, an arithmetic unit (i.e., an adder) and a comparator stage. For every atomic proposition of the ptMTL formula, one such unit is instantiated. To evaluate the hardware requirements of AtChecker units, we synthesized the respective circuits with the industrial logic synthesis tool Altera Quartus II for an Altera Cyclone IV EP4CE115 FPGA device. A single AtChecker unit consumes 290 logic elements (0.25 % of the available logic elements) and can run with a clock frequency of up to *f*_*max*_=128 MHz.

#### Example

Consider the ptMTL formula *φ*= (↑(2⋅*v*_1_+*v*_2_≤68))→(⊡_[5,10]_(4⋅*v*_3_=20∨*v*_4_=40)). Assume that the runtime verification framework is instantiated as shown in the top-right part of Fig. 1, i.e., it monitors a microcontroller core. The atomic propositions {*σ*_1_,*σ*_2_,*σ*_3_} of *φ* are: *σ*_1_≡(2⋅*v*_1_+*v*_2_≤68), *σ*_2_≡(4⋅*v*_3_=20), and *σ*_3_≡(*v*_4_=40). The symbols *v*_1_,…,*v*_4_ relate to memory locations stored in the microcontroller RAM. Together with debug information from the compiler they can be linked to high-level language symbols, e.g., C code variables. Evaluating {*σ*_1_,*σ*_2_,*σ*_2_} requires three AtChecker blocks. For example, to evaluate *σ*_1_, an AtChecker is configured to load new data from the SUT interface as soon as new values for either *v*_1_ or *v*_2_ are transferred. Its shifter is programmed to shift *v*_1_ one position to the left and the arithmetic unit so as to calculate the sum of 2⋅*v*_1_ and *v*_2_. The comparator then compares this result with the constant 68 and finally outputs the truth value of *σ*_1_ at the current time point *n*.

### Runtime observers

**Fig. 7:**
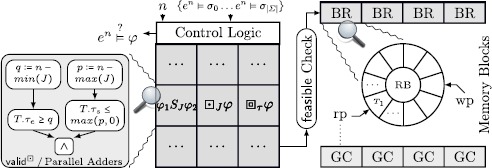
Hardware runtime observers for ptMTL specifications; abbreviations: garbage collector (GC), block ram (BR), ring buffer (RB), read pointer (rp), and write pointer (wp)

Figure 7 shows the hardware architecture to evaluate ptMTL operators. A pool of statically synthesized hardware observers is interconnected by a control logic to resemble the parse tree of the specification *φ*. For each operator we use Theorem 4 to statically assign sufficient memory to it.

#### Evaluating the observer algorithms’ predicates

Subtraction and relational operators as required by the predicates feasible, garbage, and valid can be built around adders. Observe that, when Add(〈a〉,〈b〉,c) is a ripple carry adder for arbitrary length unsigned vectors 〈a〉 and 〈b〉 and c the carry in, then a subtraction of 〈a〉−〈b〉 is equivalent to $\mathsf{Add} (\langle\mathsf{a}\rangle,\langle\overline{\mathsf{b}}\rangle, 1)$. Relational operators can be built around adders in a similar way [49, Chap. 6]. For example (left part of Fig. 7), valid ^⊡^((*τ*_*e*_,*τ*_*s*_),*n*,*J*) is implemented using five *w*-bit adders: one for *q*:=*n*−min(*J*), one for *r*:=*T*.*τ*_*e*_≥*q*, one to calculate *p*:=*n*−max(*J*) and two to calculate *t*:=*T*.*τ*_*s*_≤max(*p*,0). Finally, the unit outputs the verdict *t*∧*r*, where *t* and *r* are calculated in parallel. To evaluate  the unit uses three *w*-bit adders, one to determine *q*:=*n*−*τ*, one for *p*:=*q*>0, and a third to either calculate  or , depending on the truth value of *p*. Finally, the validity checker outputs the verdict *r* to the ptLTL evaluation unit. Note that, for the actual implementation, we do not explicitly calculate *q*:=*n*−min(*J*) through an adder. Instead, the design is configured with an absolute time point that signalizes the end of the *startup phase*, which equals to max(*J*)+1. A dedicated signal is cleared at reset and asserted once *n*=max(*J*)+1, therefore, replacing an adder by a more resource friendly comparator circuit in the implementation for the valid ^⊡^((*τ*_*e*_,*τ*_*s*_),*n*,*J*) predicate.

#### Lists and garbage collection

For a list $l_{\boxdot_{J}\varphi}$ we turn to block RAMs (abundant on contemporary FPGAs) which are organized as ring buffers (right in Fig. 7). Each ring buffer has a read (*rp*) and a write pointer (*wp*). To insert a time point pair that satisfies feasible((*τ*_*s*_,*n*−1),*n*,*J*)), *wp* is incremented to point to the next free element in the ring buffer. The GC then adjusts *rp* to indicate the latest element with regard to *n* and *J* that is *recent enough*. In a fresh cycle (indicated by a changed time point *n*), the GC loads (*τ*_*s*_,*τ*_*e*_) using *rp*, which is incremented iff garbage((*τ*_*s*_,*τ*_*e*_),*n*,*J*) holds.

#### Control logic and modularity

The control logic as shown in Fig. 7 allows one to easily reconnect hardware observers according to the specification’s parse tree, which entails that the specification can be modified (within resource limitations) without re-synthesizing the whole design, which could take tens of minutes for FPGA designs.

### A microcomputer to evaluate ptMTL and ptLTL specifications

**Fig. 8:**
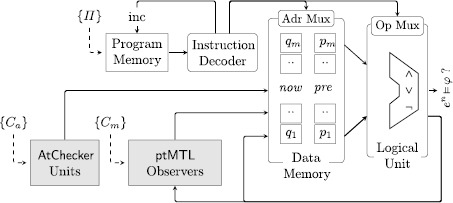
The *μ* Spy architecture. AtChecker units as in Fig. 6 and ptMTL observers as in Fig. 7

In the following, we discuss a low footprint, reconfigurable microcomputer design that uses AtChecker blocks and the hardware observer blocks to evaluate arbitrary ptLTL and ptMTL formulas. The microcomputer, called *μ* Spy, is configured with a binary program that controls and configures the building blocks depending on the formula to be evaluated. This configuration-based design of the *μ* Spy proves elegant in a dynamic setting, such as product testing in early development phases, where the specification is subject to frequent changes [70]. Modifying the specification then only requires to download a new program to the *μ* Spy. The hardware design of the *μ* Spy is shown in Fig. 8 and builds on our previous work [68, 70] where we showed how to evaluate ptLTL formulas on such an architecture. An additional component (ptMTL observers) implements the control logic needed to instantiate ptMTL hardware observers to cover the time-bounded operators of the specification.

#### Workflow

A (GUI-based) observer-generation application on a host computer compiles a ptMTL specification *φ* into a triple 〈*Π*,*C*_*a*_,*C*_*m*_〉, where *C*_*a*_ is a configuration for the AtChecker, *C*_*m*_ is a configuration for the pool of time bounded MTL operators and *Π* is a native program for the *μ* Spy.

The synthesis of a configuration for the *μ* Spy, denoted by 〈*Π*,*C*_*a*_,*C*_*m*_〉, from *φ* requires the following steps:

1. We use the ANTLR parser generator [61] to parse *φ*. This step yields an abstract syntax tree (AST) that represents the specification.
2. After some pre-processing of the AST, we determine the *m* subformulas *φ*_1_,…,*φ*_*m*_ of *φ* by using a post-order traversal.
3. For each subformula *φ*_*i*_, 1≤*i*≤*m*:
  If *φ*_*i*_ is an atomic proposition, instantiate an AtChecker block and add its configuration to *C*_*a*_.
  If *φ*_*i*_ is a ptLTL formula, we use the approach shown in [68, 70] to generate a native instruction for the *μ* Spy and add the instruction to *Π*.
  If *φ*_*i*_ is a ptMTL formula, we instantiate the corresponding observer hardware block, generate the hardware block’s configuration and a native instruction for the *μ* Spy. We add the configuration to *C*_*m*_ and the instruction to *Π*.

After running steps (1–3) of the synthesis procedure, the resulting configuration 〈*Π*,*C*_*a*_,*C*_*m*_〉 is then transferred from the host computer to the hardware platform where the *μ* Spy is instantiated on, e.g., from the host computer through an Universal Serial Bus (USB) to an FPGA. We note that the host computer is only required to generate such a configuration for the current specification, but is not required during monitoring.

#### Instruction set architecture

The *μ* Spy is a pipelined microcomputer organized as a classical Harvard architecture. Its Instruction Set Architecture (ISA) supports 22 opcodes to handle ptLTL and ptMTL operators, where each instruction word is 40 bits long. It contains the opcode, addresses of two operands, an interval address, and a further address to select a private memory space for ptMTL operators. The first two bits from the operands address denote the source of the operands data which can be a memory location, i.e., the location in the data memory where the result of the respective subformula is held, an atomic proposition or an immediate value, which can be *true* or *false*. The additional fields *Interval Address* and *List Address* are necessary for the ptMTL operators only. A single instruction word for the *μ* Spy is 40 bit long and is structured as follows:

**Table:** 

OpCode	Addr. Operand 1	Addr. Operand 2	Interval Addr.	List Addr.
5 bit	2+8 bit	2+8 bit	8 bit	7 bit

#### Architectural features

The *μ* Spy manages two memories *p*[0,…,*m*−1] and *q*[0,…,*m*−1], one containing the evaluations of all *m* subformulas of *φ* (generated in a post-order traversal of the parse tree of *φ*; in step (1) of the synthesis procedure) in the current and in the previous execution cycle (i.e., time points *n* and *n*−1). This allows for space and time efficient evaluation of formulas whose parse tree is a directed acyclic graph, and not necessarily a tree. For example, to evaluate the formula *φ*≡ (↑*σ*_1_)≡ *σ*_1_∧¬⊙*σ*_1_, one is not required to evaluate both *σ*_1_ and ⊙*σ*_1_ independently, and thus *σ*_1_ twice. Rather, we will have two registers of length 1, i.e., *p*[0] holds the result of *σ*_1_ from the previous round and *q*[0] from the current round. The *μ* Spy then fetches both *p*[0] and *q*[0] and executes the instruction that represents the operator ↑, which maps to the Boolean operation (*q*[0]⊕*p*[0])∧*q*[0], namely, *σ*_1_ did toggle its truth value (*q*[0]⊕*p*[0] holds) and *σ*_1_ is true in the current state (*q*[0] holds). Each instruction is processed through a four-stage pipeline (*fetch*, *load*, *calc*, and *write back*). All stages except the calc stage require one clock cycle per instruction, the execution time of the calc stage depends on the operator and requires from one to four clock cycles.

#### Execution time per operator

Due to the pipelined design of the *μ* Spy any ptLTL operator is executed within a single clock cycle in the pipeline stage. The additional overhead for list management and garbage collection required for the ptMTL operators require an additional one to three clock cycles. Due to a data forwarding strategy from the execution to the load stage in the pipeline, no further pipeline stalls are necessary and the pipeline is guaranteed to be optimally filled. Table 1 summarizes the execution times for various Boolean, ptLTL, and ptMTL operators.

**Table 1:** *μ* Spy clock-cycles for Boolean, ptLTL, and ptMTL operators

Logic	Operator	*μ* Spy clock cycles
Boolean	¬*φ*	1
	*φ*_0_•*φ*_1_,•∈{∧,∨}	1
ptLTL	⊙*φ*	1
	*φ*_1_ *S* *φ*_2_	1
ptMTL		2
		4
	*φ*_1_ *S*_*J*_ *φ*_2_	4

#### Example

Consider the ptMTL property *φ*≡ (↑(2⋅*v*_1_+*v*_2_≤68))→(⊡_[5,10]_(4⋅*v*_3_=20∨*v*_4_=40)). As in the example of Sect. 5.4, the atomic propositions {*σ*_1_,*σ*_2_,*σ*_3_} of *φ* are evaluated by three AtChecker units. The subformulas ↑(2⋅*v*_1_+*v*_2_≤68) and (4⋅*v*_3_=20 ∨ *v*_4_=40) are checked by the *μ* Spy. For example, the value of *σ*_1_ and the result of *σ*_1_ from time *n*−1 is used by the calc stage, which decides if ↑*σ*_1_ holds at the current time. The process is similar to determine the truth value of *σ*_2_∨*σ*_3_, the result of which is used as input to calculate ⊡_[5,10]_ (*σ*_2_∨*σ*_3_). The observer block is configured through the interval memory so as to represent *J*=[5,10]. The output of the ⊡_[5,10]_ (*σ*_2_∨*σ*_3_) calculation is then the input to the final ptLTL computation, i.e., *φ*≡ (↑*σ*_1_)→(⊡_[5,10]_(*σ*_2_∨*σ*_3_)).

## Evaluation

To demonstrate the feasibility of our approach, we implemented the presented algorithms for ptMTL monitoring by means of the *μ* Spy on an FPGA platform. In the current implementation, subformulas are evaluated sequentially as they appear in the specification’s parse tree. Since the observer blocks are executed in sequence, their logic elements can be reused and it suffices to equip the *μ* Spy with only one , one ⊡_*J*_ *φ*, and one *φ*_1_ *S*_*J*_ *φ*_2_ hardware observer block and assign memory according to the number of subformulas.^2^ The implementation is a synchronous register-transfer-level VHDL design, which we both simulated in Mentor Graphics ModelSim and synthesized for various FPGAs using the industrial logic synthesis tool Altera Quartus II.^3^

### Simulation results

We conducted several simulation runs of the VHDL implementation of the *μ* Spy unit when monitoring different ptMTL formulas with randomly generated inputs, representing the execution traces of an SUT. The simulation runs cover several combinations of the ptLTL operators ↑, ⊙, and *φ*_1_ *S*_*s*_ *φ*_2_ as well as the time-bounded ptMTL operators , , and *φ*_1_ *S*_*J*_ *φ*_2_. The truth values of the involved atomic propositions {*σ*_0_,*σ*_1_,*σ*_2_} were generated by placing 1000 truth value transitions with uniformly distributed interarrival times on the discrete timeline. In all simulated executions, our implementation behaved as specified. To increase confidence in the implementation, we used an automatic test suite, which checks the generated executions not only with the *μ* Spy, but also with (i) a software implementation of our observer algorithms and (ii) a naive offline monitoring algorithms following the semantics definition of ptLTL and ptMTL. We run this setup with a set of sample specifications and compared the output of the three implementations and iteratively fixed remaining bugs. We used traditional line coverage metrics to assess the test progress. A rigorous, formal correctness analysis of the *μ* Spy implementation, however, is still an open issue.

In what follows, we discuss two representative simulation runs involving the  and the *S*_*J*_ operator. To make the simulation traces accessible, Table 2 summarizes all relevant hardware signals and their intended meaning. We further use the following annotation for the internal state of the *μ* Spy: *m*(*x*) denotes the location in the observer’s data memory at address *x*, *a*(*x*) denotes the *x* ^*th*^ atomic proposition and *i*(*x*) specifies the interval stored at address *x* in the observer’s interval memory.

**Table 2:** Simulation signals and their meaning; *AH* = *Active High* (issued when high); *AL* = *Active Low* (issued when low), and *RTC* = *Real Time Clock*

Signal Name	Unit	Meaning
s_clk	RVU	system clock of the RV framework
s_reset_n		asynchronous reset of the RV framework (AL)
s_sut_clk		system clock of the SUT
s_rtc_timestamp	RTC	ctr. value of the real-time clock (i.e., time point *n*)
s_atomic(0)	SUT	truth value of atomic proposition # 0, *σ*_0_ (AH)
s_atomic(1)		truth value of atomic proposition # 1, *σ*_1_ (AH)
s_atomic(2)		truth value of atomic proposition # 2, *σ*_2_ (AH)
s_atomic(3)		truth value of atomic proposition # 3, *σ*_3_ (AH)
s_violated	RVU	monitoring output *e* ^*n*^⊨*φ* (AH)
command	*μ* Spy	instruction (op-code) for the *μ* Spy
state		state of the fetch stage state machine
state		state of the load stage state machine
state		state of the calc stage state machine
state		state of the write back stage state machine
interval_min		min(*J*) (in RTC ticks)
interval_max		max(*J*) (in RTC ticks)
sel	List	select the list specified by buffer_nr (AH)
add_start		add start () time point to the list (AH)
add_end		add end () time point to the list (AH)
set_tail		clear list and add new entry (AH)
reset_tail		clear list and add entry with time point 0 (AH)
drop_tail		remove tail element from the list (AH)
delete		remove head element from the list (AH)
buffer_nr		id of the currently used list (AH)

#### (a) Invariant previously 

We setup the framework so as to evaluate the ptMTL formula: 
 The property is then translated by the host application into the following binary program for the *μ* Spy:

01011 0000000000 0000000000 00000000 0000000 // rising edge at a(0)

10001 0000000001 0000000000 00000001 0000000 // [[]] a(1), i(1), mem 0

00110 1000000000 1000000001 00000000 0000000 // m(0) -> m(1)

11111 1000000010 0000000000 00000000 0000000 // output result m(2)

and into the following data for the interval memory:

0000000000000000 0000000000000110 // startup phase duration: 6

0000000000000000 0000000000000101 // [0, 5]

The binary program consists of three subformulas and a dedicated *end* instruction. The interval memory holds two entries, the first denotes the duration of the start-up phase in RTC clock cycles and the second entry holds *τ*=5 for the  operator. The startup phase signal is then used to implement the check whether *n*−*τ*≥0 in the  predicate.

**Fig. 9:**
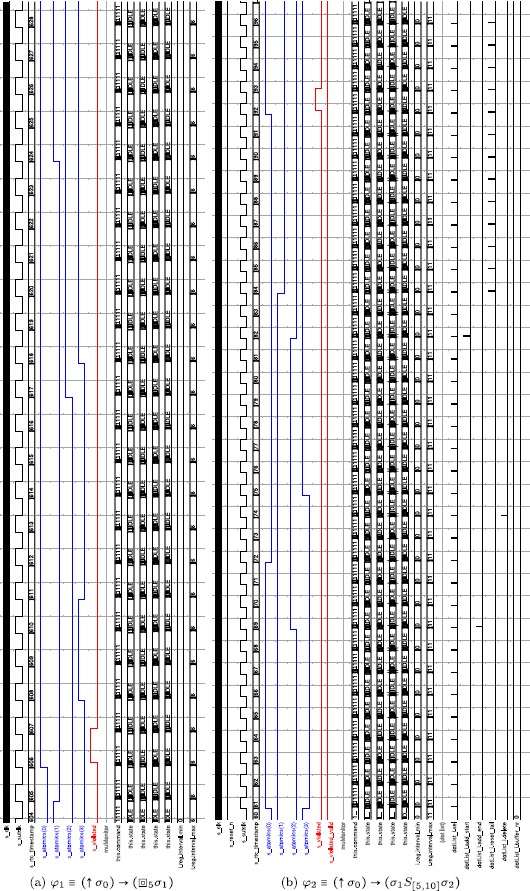
Simulation traces for *φ*_1_ and *φ*_2_ extracted from ModelSim

The simulation screenshot in Fig. 9a shows a section of the simulated VHDL entities. At time point *n*=606, we see a  transition of s_atomic(0) which makes the premise of the implication true. As s_atomic(1) does not hold for all times within the interval [601,606],  and the implementation correctly asserts the violated signal. According to Algorithm 1, the next  transition of s_atomic(1) at time *n*=617 is stored in the  memory of the  operator. At the next  transition of s_atomic(0) at time *n*=624 the premise of the implication holds and  is evaluated as follows: 624−5≥617, yielding true, thus, *e* ^624^⊨*φ*_1_.

#### (b) Since within interval *φ*_1_ *S*_*J*_ *φ*_2_

We setup the framework so as to evaluate the ptMTL formula:

$$\varphi_2 \equiv\,(\uparrow\sigma_0) \rightarrow ( \sigma_1\; S_{[5,10]}\;\sigma_2) $$

The property is then translated by the host application into the following binary program for the *μ* Spy:

01011 0000000000 0000000000 00000000 0000000

        // rising edge at a(0)

10011 0000000001 0000000010 00000001 0000000

        // a(1) S a(2), i(1), mem 0

00110 1000000000 1000000001 00000000 0000000

        // m(0) -> m(1)

11111 1000000010 0000000000 00000000 0000000

        // output result m(2)

and into the following data for the interval memory:

0000000000000000 0000000000001011

        // startup phase duration: 11

0000000000000101 0000000000001010 // [5, 10]

The instruction memory contains three instructions corresponding to the three operators in the formula. Figure 9b shows a snippet of the corresponding simulation trace. At time *n*=69 a  transition of s_atomic(2) is detected and according to Algorithm 3, *n*−1=68 is added to the list *l*_*S*_ of the *S* observer which is triggered by the *add_end* signal. At time *n*=74 the predicate garbage evaluates to true (since (68<74−min(5,10)) holds) and triggers the deletion of the element in the list. The signal *delete* is asserted. The  transition of s_atomic(2) at time *n*=82 triggers the adding of the interval-start time point to *l*_*S*_ (see Algorithm 3 line 4). Consequently (82,∞) is the new head element of *l*_*S*_. Starting from time *n*=84 on s_atomic(1) and s_atomic(2) are false, which, according to Algorithm 3, sets the list to (0,∞). This is done through the *reset_tail* signal. At time *n*=92 we see a  transition of s_atomic(0) which yields *e* ^92^⊨ (↑*σ*_0_). The valid ^⊡^ predicate evaluates as follows: (0≤92−max(5,10))∧(∞≥92−min(5,10)), yielding true. Finally, we obtain $e^{92} \not\models\varphi_{2}$ and the *violated* signal is asserted.

### Performance study

Recall, that our hardware implementation uses one hardware module for  and  observers, one for the ⊡_*J*_ *φ* and  observers, and one for *φ*_1_ *S*_*J*_ *φ*_2_ observers. The latter two modules both require lists of the same size, therefore, scale identically with respect to operating frequency, logic elements, and required memory size. We thus treated them equally within the performance study.

A hardware instantiation of the *μ* Spy with the standard configuration of 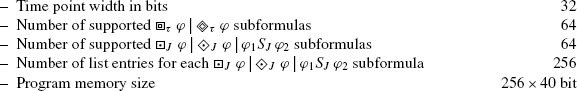
requires a total of 1297 logic elements and 1.075.392 memory bits (132 kByte) and allows for a maximum operating frequency *f*_*max*_ of 106 MHz (for the slow timing model at 85 ^∘^C) on an Altera Cyclone IV EP4CE115 FPGA. The operating frequency can easily be increased by moving to a more powerful FPGA architecture. For example, when synthesizing the design for an Altera Stratix V FPGA, we obtain a maximum operating frequency *f*_*max*_ of 230 MHz.

#### Scalability

**Fig. 10:**
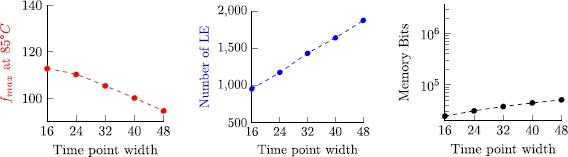
Maximum operating frequency *f*_*max*_, number of logic elements (LE), and required memory bits versus time point width, assuming a fixed number of 64 subformulas of type  and 1 of type

**Fig. 11:**
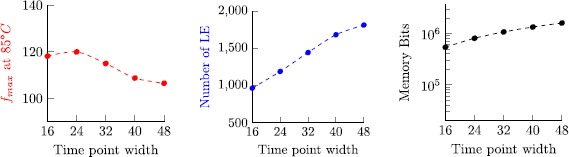
Maximum operating frequency *f*_*max*_, number of logic elements (LE), and required memory bits versus time point width, assuming a fixed number of 1 subformula of type  and 64 of type

We synthesized the *μ* Spy with different parameters to assess its scalability with regard to the width of the time points as well as the maximum number of ptMTL subformulas supported by the *μ* Spy. We ran the synthesis with default settings so as to not obscure measurements by tool-specific optimizations. For example, when running synthesis optimized for speed, we naturally obtained results with higher operating frequencies but also with a higher number of logic elements. The influence of the *time point width* on the synthesized designs is shown in Figs. 10 and 11. Both figures show the scalability of the operating frequency, number of logic elements and required memory bits with respect to time point width. To asses scalability of each of the observer modules we built one variant supporting 64 (respectively 1) subformula(s) of type  and 1 (respectively 64) subformula(s) of type . For both design variants, operating frequency and required number of logic elements scale linearly with comparable slope. For example, doubling the time point width from 24 to 48 bit increases the number of logic elements by about 60 %, whereas we observe only a 13 % decrease in the achievable maximum operating frequency. However, the design is still considerably small. Even with a time point width of 48 bit, it requires only 1.4 % of the available logic elements on our (low-end) target FPGA. For the number of required memory bits we observe a significant difference for both variants: Since the hardware module for evaluating  operators is equipped with a memory to store a list of time points for each of the supported  subformulas, the required memory bits increase significantly faster in the variant supporting 64 such subformulas than in the version supporting only a single such subformula.

**Fig. 12:**
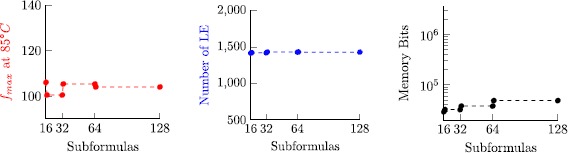
Maximum operating frequency *f*_*max*_, number of logic elements (LE), and required memory bits versus number of subformulas of type , assuming a fixed number of 1 subformula of type  and time point width 32

**Fig. 13:**
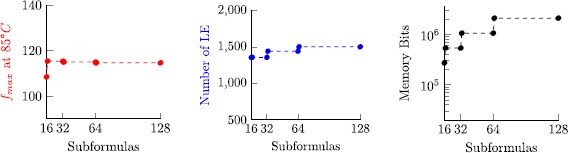
Maximum operating frequency *f*_*max*_, number of logic elements LE, and required memory bits versus number of subformulas of type , assuming a fixed number of 1 subformula of type  and time point width 32

Figures 12 and 13 show the influence of the *number of supported subformulas* of type  and of type . For that purpose, we built variants supporting a varying number of subformulas of type  and only one subformula of type , and vice versa. One immediately sees that the number of supported subformulas of both types is not a limiting factor with respect to operating frequency and number of logic elements, as both stay almost constant. This is mainly due to the design choice we made for the *μ* Spy, where we implemented the predicates, checks, and control logic required to evaluate either , , or *φ*_1_ *S*_*J*_ *φ*_2_ subformulas only once and reuse this hardware blocks every time the *μ* Spy executes an opcode for a time-bounded subformula. To put this results in perspective, trimming the design of the *μ* Spy to evaluating ptLTL specifications only accounts for 294 logic cells (23 % of the original design) and an *f*_*max*_ of 122 MHz (114 % of the original design). The situation is different for the required memory. It increases significantly with the number of supported subformulas: For each additional supported subformula, a sufficiently large memory block has to be added to the design. Clearly this leads to larger increases for subformulas that require to store lists of time points (cf. Fig. 13) than those that require to store only a single time point (cf. Fig. 12).

## Related work

This section surveys related work by focusing on frameworks and tools, theoretical results on observer algorithms, and approaches that perform runtime verification either in or of hardware designs.

### Frameworks and tools

Watterson and Heffernan [80] review established and emerging approaches for monitoring (software) executions of embedded systems; calling for future work on runtime verification approaches that utilize existing chip interfaces to provide the observations as events to an external monitoring system. Pike et al. [64] worked on runtime verification for real-time systems by defining observers in a data-flow language, which are compiled into programs with constant runtime and memory. If the original system is periodically schedulable with some safety margin, the monitored system can be shown to be schedulable, too. This approach targets software only, whereas we monitor a combination of embedded software and hardware components. Hardware observers that simply probe one or more internal signals have been known in literature for a few decades. An early instance thereof is the non-interference monitoring and replay mechanism by Tsai et al. [79]. Their monitoring system is based on the MC6800 processor that records the execution history of the target system. A dedicated replay controller then replays stored executions, which supports test engineers in low-level debugging. Although we share a similar idea of probing internal signals, our framework detects specification violations on-the-fly, rather than replaying traces from some execution history.

The Dynamic Implementation Verification Architecture (DIVA) exploits runtime verification at intra-processor level [5]. Whenever a DIVA-based microprocessor executes an instruction, the operands and the results are sent to a checker which verifies correctness of the computation; the checker also supports fixing an erroneous operation. Chenard [19] presents a system-level approach to debugging based on in-silicon hardware checkers. The work of Brörkens and Möller [18] is akin to ours in the sense that they also do not rely on code instrumentation to generate event sequences. Their framework, however, targets Java and connects to the bytecode using the Java Debug Interface (JDI) so as to generate sequences of events.

BusMOP [62] generates observers for ptLTL on FPGAs, which are connected to the Peripheral Component Interconnect (PCI). The commercial Temporal Rover system [29] implements observers for MTL formulas, but the implementation and algorithms used are not published.

### Observer algorithms

We restrict our survey to ptMTL observer algorithms for past time logics in the discrete-time setting.

Thati and Roşu [78] presented an on-line observer for MTL formulas *ψ*. Their idea is to reduce the problem of deciding whether *e* ^*n*^⊨*ψ* to deciding several instances of *e* ^*n*′^⊨*ψ*′, where *ψ*′ is a subformula of *ψ* and *n*′≤*n*. Thereby for each subformula *φ*_1_ *S*_[*a*,*b*]_ *φ*_2_ of *ψ*, the formulas *φ*_1_ *S*_[*a*−1,*b*−1]_ *φ*_2_, *φ*_1_ *S*_[*a*−2,*b*−2]_ *φ*_2_, …, *φ*_1_ *S*_[0,*b*−*a*]_ *φ*_2_, …, *φ*_1_ *S*_[0,0]_ *φ*_2_ are defined to be subformulas of *ψ*. For example, in case *ψ*≡*φ*_1_ *S*_[1,3]_ *φ*_2_, where *φ*_1_ and *φ*_2_ are atomic propositions, the reduced formulas of *ψ* are *φ*_1_, *φ*_2_ as well as *φ*_1_ *S*_[0,2]_ *φ*_2_, *φ*_1_ *S*_[0,1]_ *φ*_2_, and *φ*_1_ *S*_[0,0]_ *φ*_2_. Denoting by *m* the number of subformulas an MTL formula *ψ* is reduced to, the space complexity of their observer is within $\mathcal{O}(m 2^{m})$ and its time complexity is within $\mathcal{O}(m^{3} 2^{3m})$ for each time *n* in $\mathbb{N}_{0}$, the observer is executed. For the special cases of *ψ*≡*φ*_1_ *S*_*J*_ *φ*_2_, the observer still requires a memory of at least 2*m*≥2max(*J*) bit. While this bound is incomparable in general to our bound, for large values of max(*J*) we immediately obtain that our solution has less memory complexity. For example for *φ*_1_ *S*_[5,1500]_ *φ*_2_ the solution in [78] requires at least 3000 bit of memory, whereas our observer requires 208 bit, assuming (upper bounded) time points of 52 bit.

Maler et al. [57] presented an on-line observer algorithm for *φ*_1_ *S*_*J*_ *φ*_2_ that is based on having active counters for each event of *φ*_2_. Divakaran et al. [26] improved the number of counters of bit width logmax(*J*) to $2\lceil\min(J)/(\operatorname{len}(J))\rceil+ 2$ and proved that any Since observer realized as a timed transition system must use at least $2(\lceil\min(J))/(\operatorname{len}(J))\rceil+ 1$ clocks. While their space complexity is incomparable to ours in general, their solution is very resource intensive for a hardware realization: While we may store list values in cheap RAM blocks, their solution requires to store the current counter values in registers, since their values are incremented at every time step. Further, one can show by simple algebraic manipulations that:

### Proposition 2

*For all intervals J*, *with* 0≤min(*J*)<max(*J*)<∞,

9 $$ \frac{2\max(J)-\min(J)+2}{2+\operatorname{len}(J)} - \frac{\min (J)}{\operatorname{len}(J)} \le2. $$

### Proposition 3

*For all intervals J*=[*a*,*a*+1], *where* $a \in\mathbb{N}_{0}$,

$$ \frac{\min(J)}{\operatorname{len}(J)} - \frac{2\max(J)-\min (J)+2}{2+\operatorname{len}(J)} = \frac{2}{3} (a-2 ) . $$

From Proposition 2 immediately follows that our observer requires at most two tuples in addition to the (counter) tuples required by Divakaran et al.’s observer. On the other hand, it follows from Proposition 3 that there exists a choice of parameters where our observer requires significantly less memory.

In contrast to the solution presented by Divakaran et al. [26], our solution is tailored to a discrete time base, dictated by our application domain: not only that at the hardware level a (discrete) system clock is naturally available, but also adding and comparing fractions would incur a significant overhead with respect to latency and circuit size. Nonetheless, our algorithms also work in the dense time domain with only two small modifications: (i) instead of running the algorithms at every time $n\in\mathbb{N}_{0}$, they need to be executed at every transition of an input signal, and (ii) the term “*n*−1” must be replaced by “*n*” in Algorithms 2 and 3. By analogous proofs we obtain that, in this case, list *ℓ* is of size at most $(\max(J))/(\operatorname{len}(J))+1$ tuples, which is at most one more than the number of clocks required by the Since observer by Divakaran et al. [26].

Basin et al. [11] present a (discrete time) point-based observer for formula *φ*_1_ *S*_*J*_ *φ*_2_ which runs in time $\mathcal{O}(\log\max(J\cup\{n\}))$ if executed at time $n\in \mathbb{N}_{0}$. Their algorithm, however, requires memory in the order of max(*J*). They further presented an interval-based observer algorithm for *φ*_1_ *S*_*J*_ *φ*_2_ with space complexity comparable to our solution. However, the algorithm is clearly motivated with a software implementation in mind, whereas we aim at efficient (highly parallel) circuit implementations. For example, for an arbitrary ptMTL formula *φ*, our time-complexity bounds scale with the depth of the parse tree of *φ*, in case the *μ* Spy executes observer algorithms in parallel, and with the number of nodes in the parse tree of *φ*, in case the *μ* Spy executes observer algorithms sequentially. By contrast, the bounds in [11] scale with the fourth power of the number of nodes in the parse tree of *φ*. Further, a direct implementation of their algorithm would require considerable hardware overhead, as it makes use of doubly-linked lists to store and manipulate time points. In comparison, our ring buffer design can easily be mapped to block RAM elements that are abundant on modern day FPGAs.

### Hardware observers

In previous work, we have shown that ptLTL can, within certain bounds, be checked in hardware running at the same frequency as the SUT [68]. Assertion-based verification (ABV) [36] gained momentum in industrial-strength hardware verification, especially driven by the emerge of the Property Specification Language (PSL). PSL is based on LTL, augmented with regular expressions, thus, we will not compare our work to PSL monitoring algorithms but rather to the hardware architecture of the resulting checkers. Existing work largely aims at synthesizing hardwired circuits out of various temporal specifications, whereas our approach (a) focuses on ptMTL specifications and (b) aims at providing a reconfigurable framework that has also applications in testing and not only as hard-coded observer. Translations from PSL into hardware either follow the modular or the automata based synthesis.

In the modular approach [14, 15, 25, 27, 60], sub-circuits for each operator are built and inter-connected according to the parse tree of the PSL expression being monitored. These circuits then output a pair of signals indicating the status of the assertion. Boulé and Zilic [15] present a hardware-checker generator capable of supporting ABV, by translating PSL to hardware language descriptions that can be included into the source design. The input to their circuit generator is the source file of the design under test (DUT). This limits their approach to designs where the source is available, whereas our framework can be attached to a variety of targets (cp. Fig. 1), even third party proprietary systems. Unfortunately, their algorithms lack a complexity analysis. Borrione et al. [14] describe a method of translating properties of the PSL foundation layer into predefined primitive components. A component is a hardware unit, consisting of a checking window and an evaluation block. They make use of shift register chains in the checking window block to trigger the execution of the evaluation block. Primitive components representing a timed operator (e.g., within in the next *τ* time units), need to individually count the number of elapsed time points. Das et al. [25] presented a modular approach by decomposing System Verilog Assertions (SVA) into simple communicating parallel hardware units that, when connected together, act as an observer for a SVA. Morin-Allory and Borrione [60] describe a generation of synthesizable hardware from regular expressions included in PSL. Drechsler [27] describes an approach to synthesize checkers for online verification of SoC designs through chains of shift registers, but does not allow for checking arithmetic relations among bit-vectors. For hardware designs, these specifications are often directly available from the specification [75].

In the automata based approach [4, 16, 17, 37, 38, 56], state machines are synthesized that check a property during simulation. The generated automata are generally of non-deterministic nature. To avoid a blowup of the automaton capable of monitoring formulas that are required to hold for a certain number of clock cycles, additional counters are inserted. However, this is only feasible if the output language natively supports non-deterministic finite automata (NFA), unfortunately, major hardware descriptions languages (e.g., Verilog and VHDL) do not. Consequently, observers need to be converted to a deterministic finite automaton (DFA) first, which, in the worst case, yields an exponential blowup of the resulting DFA in the size of the NFA [43]. This theoretical limitations were also reflected in the experiments of Straka et al. [76] where they report on an attempt to verify trivial properties of a simple counter, where the resulting observers synthesized by FoCs [1] from a PSL specification requires 120 logic slices whereas the resources for the counter itself accounts only for 3 slices. This performance issues motivate them to turn to a self-made tool to design on-line checkers instead of using existing toolchains. Lu and Forin [56] present a compiler from Psl to Verilog, which translates a subset of Psl assertions (sPsl, a C-language binding for Psl [20]) about a software program (written in C in their approach) into hardware execution blocks for an extensible MIPS processor, thus allowing for transparent runtime verification without altering the program under investigation. The synthesized verification unit is generated by a property rewriting algorithm developed by Roşu and Havelund [72]. Atomic propositions are restricted to a single comparison operator only. For comparison, our approach supports more complex relations among memory values in the atomic propositions, thus yielding greater flexibility and expressiveness in the specification language. Armoni et al. [4] describe an automata-theoretic construction based on determinization for unrestricted temporal logic, i.e., ForSpec [3]. They showed how to obtain deterministic compilation targeting dynamic verification that is as close as possible to the nondeterministic compilation of temporal assertions.

## Conclusion

We presented an on-line runtime verification framework to check a ptMTL formula on executions with discrete time domain. At the framework’s heart is an observer design for the time-bounded Since operator and the special cases of *exists*/*invariant previously* and *within interval*. Correctness proofs of all presented algorithms have been given and bounds on their time and space complexity have been proven. The promising complexity results are mainly due to the integration of a garbage collection and a filtering strategy that automatically drop events that can neither validate nor invalidate the specification.

We further discussed a reconfigurable hardware realization of our observer algorithm that provides sufficient flexibility to allow for changes of the monitored specification without necessarily re-synthesizing the hardware observer. Reconfigurability is indeed a valuable property of the presented approach since logic synthesis is itself a very time-consuming task. To demonstrate the feasibility of our approach for practical applications, we implemented the algorithms on a Field Programmable Gate Array. The predictable and low resource requirements of the presented hardware solution together with its reconfigurability support the application in the diagnosis of embedded real-time systems during execution time.

Based on the framework presented in this article, we plan to investigate the following directions: *who guards the guardians?* [74] is a legitimate question with regard to the implementation of our runtime verification unit. Whereas we gave a formal correctness analysis for the algorithms itself, however, doing so for the implementation is an open issue. Additionally, we plan to extend our work to (bounded) future time MTL specifications.

## Footnotes

1 In our framework, we thus assume time points to be from $\mathbb{N}_{0}$.

2 In our experiments, we opted for a resource efficient design of the *μ* Spy. A configuration of the *μ* Spy with multiple ptMTL hardware observers immediately makes an evaluation of several subformulas in parallel possible, however, increase resource requirements.

3 Tools can be downloaded from http://www.mentor.com and http://www.altera.com.
